# Glycemic control in critically ill patients with or without diabetes

**DOI:** 10.1186/s12871-022-01769-4

**Published:** 2022-07-16

**Authors:** Ka Man Fong, Shek Yin Au, George Wing Yiu Ng

**Affiliations:** 1 Department of Intensive Care, Queen Elizabeth Hospital, 30 Gascoigne Road, Kowloon, Hong Kong; 2 Department of Medicine, Tseung Kwan O Hospital, Tseung Kwan O, Hong Kong

**Keywords:** Blood glucose, Electronic health record, Hypoglycemia, Hyperglycemia, ICU, Insulin therapy, Diabetes, Stress-induced hyperglycemia

## Abstract

**Background:**

Early randomized controlled trials have demonstrated the benefits of tight glucose control. Subsequent NICE-SUGAR study found that tight glucose control increased mortality. The optimal glucose target in diabetic and nondiabetic patients remains unclear. This study aimed to evaluate the relationship between blood glucose levels and outcomes in critically ill patients with or without diabetes.

**Methods:**

This was a retrospective analysis of the eICU database. Repeat ICU stays, ICU stays of less than 2 days, patients transferred from other ICUs, those with less than 2 blood glucose measurements, and those with missing data on hospital mortality were excluded. The primary outcome was hospital mortality. Generalised additive models were used to model relationship between glycemic control and mortality. Models were adjusted for age, APACHE IV scores, body mass index, admission diagnosis, mechanical ventilation, and use of vasopressor or inotropic agents.

**Results:**

There were 52,107 patients in the analysis. Nondiabetes patients exhibited a J-shaped association between time-weighted average glucose and hospital mortality, while this association in diabetes patients was right-shifted and flattened. Using a TWA glucose of 100 mg/dL as the reference value, the adjusted odds ratio (OR) of TWA glucose of 140 mg/dL was 3.05 (95% confidence interval (CI) 3.03–3.08) in nondiabetes and 1.14 (95% CI 1.08–1.20) in diabetes patients. The adjusted OR of TWA glucose of 180 mg/dL were 4.20 (95% CI 4.07–4.33) and 1.49 (1.41–1.57) in patients with no diabetes and patients with diabetes, respectively. The adjusted ORs of TWA glucose of 80 mg/dL compared with 100 mg/dL were 1.74 (95% CI 1.57–1.92) in nondiabetes and 1.36 (95% CI 1.12–1.66) in patients with diabetes. The glucose ranges associated with a below-average risk of mortality were 80–120 mg/dL and 90–150 mg/dL for nondiabetes and diabetes patients, respectively. Hypoglycemia was associated with increased hospital mortality in both groups but to a lesser extent in diabetic patients. Glucose variability was positively associated with hospital mortality in nondiabetics.

**Conclusions:**

Time-weighted average glucose, hypoglycemia, and glucose variability had different impacts on clinical outcomes in patients with and without diabetes. Compared with nondiabetic patients, diabetic patients showed a more blunted response to hypo- and hyperglycemia and glucose variability. Glycemic control strategies should be reconsidered to avoid both hypoglycemia and hyperglycemia.

**Supplementary Information:**

The online version contains supplementary material available at 10.1186/s12871-022-01769-4.

## Introduction

Hyperglycemia is frequently observed in critically ill patients, and it has long been viewed as an adaptive response to acute stress that provides energy to the nervous system and immune system [1, 2]. However, the landmark Leuven I study published in 2001 showed mortality benefits with tight glycemic control at 80–110 mg/dL compared with liberal glycemic control at 180–200 mg/dL in surgical patients admitted to intensive care units (ICUs) [3]. The practice of tight glycemic control was widely adopted until 2009, when the pendulum swung to favor an intermediate glucose target at 140–180 mg/dL after the publication of the NICE-SUGAR study in which tight glucose control resulted in a higher risk of hypoglycemia and mortality than intermediate glucose control (140–180 mg/dL), in patients from medical-surgical ICUs [4]. As the largest randomized control trial in this field to date, the NICE-SUGAR study appeared to offer strong evidence against tight glucose control in critically ill patients. However it has been argued that methodological flaws in the protocol in terms of glucose measurement and insulin administration resulted in an excessive incidence of hypoglycemia [5]. Furthermore, it remains unclear whether patients with different characteristics might benefit from individualized glucose targets. Studies have shown that diabetic patients seem to respond differently to glycemia than nondiabetic patients, and thus, the concept of personalized glucose targets has been advocated [6, 7]. This was first evidenced by the lack of benefit of tight glucose control in diabetic patients in the post hoc analysis of Leuven I and II [8]. A higher glucose range was associated with lower mortality in patients with higher preadmission HbA1c levels [9]. Therefore, there has been a keen interest in searching for a higher glucose range for diabetic patients. However, the results from the 2 before-and-after studies evaluating liberal glucose targets by Krinsley et al. and Luethi et al. were inconsistent [10, 11]. A randomized controlled trial has been attempted in order to individualize glucose targets based on HbA1c levels, but the study was prematurely terminated because of the low likelihood of benefit and the risk of hypoglycemia [12]. Intensivists remain unclear about the optimal target, and a significant variation in glycemic control practices across centers has been observed [13, 14].

The aim of this study was to evaluate the relationship between blood glucose levels and outcomes in critically ill patients with or without diabetes. It was postulated the optimal glucose target for diabetes patients might differ from that for nondiabetes patients.

## Methods

### Data description

The eICU Collaborative Research Database was chosen for data analysis. It is a free, deidentified, multicenter ICU database including 200,859 ICU admissions in the period of 2014–2015 accounted for by 139,369 unique patients at 208 hospitals throughout the U.S. [15, 16]. All the data have been deidentified to meet the safe harbor provision of the US Health Insurance Portability and Accountability Act (Certification no. 1031219–2). We excluded repeat ICU stays, ICU stays of less than 2 days, patients transferred from other ICUs, patients with fewer than 2 blood glucose measurements, patients who were admitted for diabetic ketoacidosis (DKA) or diabetic hyperglycemic hyperosmolar nonketotic coma (HHNC), and cases with missing data on hospital mortality. Diagnoses of diabetes were identified from patients’ past histories and medications. HbA1c data were not available from the eICU database. Blood glucose measurements including laboratory measurements and bedside testing were included. Patients admitted for trauma were identified by the APACHE diagnosis. This study was reported in accordance with the Strengthening the Reporting of Observational Studies in Epidemiology statement [17].

The primary outcome of this study was hospital mortality. Secondary outcomes included occurrences of severe hypoglycemia (≤40 mg/dL) and moderate hypoglycemia (41–70 mg/dL) [18].

### Statistical analysis

Glucose measurements were reported with reference to the consensus recommendations by Finfer et al. [18]. For central tendency, we reported the time weighted average (TWA) glucose level during the ICU stay in addition to the mean glucose level to avoid bias from unequal time measurements [19]. (See supplementary Table 1 for the formula for TWA glucose) For glycemic variability, we reported both the standard deviation and coefficient of variation (CV) to facilitate comparisons with previous studies (See supplementary Table 1 for the CV formula). The minimum glucose level and the rates of severe hypoglycemia (≤40 mg/dL) and moderate hypoglycemia (41–70 mg/dL) were reported.

Generalized additive models (GAMs) were employed to evaluate relationship between each domain of glycemic control and hospital mortality in diabetes and nondiabetes patients. Confounding factors including age, Acute Physiology and Chronic Health Evaluation (APACHE) IV scores, body mass index (BMI), admission diagnosis (surgical vs. medical), mechanical ventilation, and use of vasopressor or inotropic agents during ICU stays were adjusted. BMI was included as a higher BMI has recently been reported to be a potentially protective factor against hypoglycemia and mortality [20]. GAM allows the modeling of nonlinear relationships between independent variables and outcomes [21, 22]. The partial effect of the continuous independent variables was presented in terms of the probability of mortality. The odds ratio (OR) for the specific increment in continuous variables was calculated. Subgroup analyses were performed for patients admitted for medical and surgical diagnoses, and patients admitted for trauma and nontrauma. Sensitivity analysis was carried out in nondiabetes and diabetes patients grouped by their therapies, as follows: insulin-dependent diabetes (IDDM), non-insulin-dependent diabetes on oral hypoglycemic agents (NIDDM on OHA), and non-insulin-dependent diabetes on diet (NIDDM on diet). Sensitivity analysis was also carried out to include all ICU patients, including those who stayed in the ICU for less than 2 days. Furthermore, considering the censoring nature of ICU discharge, the Cox proportional hazard model with glucose as the time-varying covariate was performed to explore the time-varying effect of glucose on ICU mortality. Schoenfeld’s residual test was used to test for the violation of proportional assumptions [23–25].

Continuous variables are presented as the medians with interquartile ranges, and categorical variables are presented as numbers with percentages. Differences between groups were assessed using the Mann–Whitney U test, the chi-square test and Fisher’s exact test as appropriate. Data cleaning and preprocessing were performed using Python (version 3.8.2). We used R (version 4.1.2, The R Foundation for Statistical Computing) to perform statistical modeling. GAM was performed using the mgcv package v1.8–38 [26, 27]. The OR of the GAM was calculated using the oddsratio package v1.0.2 [28]. All statistical tests were two-sided, and a *p* value < 0.05 was considered significant.

The source code for the analyses can be found at https://github.com/ckmfong/glucose.

## Results

Supplementary Figure 1 shows the inclusion of 52,107 patients in the analysis. Table 1 tabulates the patient demographics, glycemic control and outcomes of nondiabetes and diabetes patients. Compared with nondiabetes patients, patients with diabetes were older and had higher BMI and APACHE IV scores. They were also more often admitted for medical diagnoses. Diabetes patients more often required mechanical ventilation and vasopressors or inotropic agents. Patients with diabetes also had higher ICU and hospital mortality. Supplementary Table 2 shows the number of nondiabetes patients and patients with diabetes on insulin, oral hypoglycemic agents, or diet. Supplementary Table 3 lists the top ten diagnoses for patients admitted for medical or surgical diagnoses.

**Table 1:** Patient demographics, glycemic control and outcomes of nondiabetes and diabetes patients

	All (*n* = 52,107)	No diabetes (*n* = 36,455)	Known diabetes (*n* = 15,652)	*p*-value
Age (years) ^a^	67.0 (55.0–77.0)	65.0 (53.0–77.0)	68.0 (59.0–77.0)	< 0.001
Male ^a^	28,513 (54.7)	19,949 (54.7)	8564 (54.7)	0.996
BMI (kg/m^2^) ^a^				
< 18.5	4592 (8.8)	3840 (10.5)	752 (4.8)	< 0.001
18.5- < 25	13,211 (25.4)	10,148 (27.8)	3063 (19.6)	
25- < 30	11,583 (22.2)	8294 (22.8)	3289 (21.0)	
30- < 35	8762 (16.8)	5853 (16.1)	2909 (18.6)	
35- < 40	5392 (10.3)	3352 (9.2)	2040 (13.0)	
≥ 40	6900 (13.2)	3792 (10.4)	3108 (19.9)	
APACHE IV ^a^	59.0 (44.0–78.0)	58.0 (42.0–76.0)	63.0 (48.0–81.0)	< 0.001
Admission diagnosis ^a^				< 0.001
Medical	41,387 (79.4)	28,655 (78.6)	12,732 (81.3)	
Surgical	10,294 (19.8)	7394 (20.3)	2900 (18.5)	
Elective	8813 (16.9)	6253 (17.2)	2560 (16.4)	
Emergency	1481 (2.8)	1141 (3.1)	340 (2.2)	
Mechanical ventilation ^a^	18,786 (36.1)	12,960 (35.6)	5826 (37.2)	< 0.001
On inotropes or vasopressors	12,509 (24.0)	8489 (23.3)	4020 (25.7)	< 0.001
Trauma	2576 (4.9)	2229 (6.1)	347 (2.2)	< 0.001
On steroid	9543 (18.3)	6673 (18.3)	2870 (18.3)	0.942
Glycemic control				
TWA blood glucose (mg/dL)^b^	125.9 (109.4–152.1)	118.0 (105.5–134.5)	158.1 (133.3–186.4)	< 0.001
Mean blood glucose (mg/dL)	127.4 (110.9–152.1)	120.0 (106.8–136.1)	156.8 (133.0–183.8)	< 0.001
Mean laboratory blood glucose (mg/dL)	125.7 (109.0–151.5)	118.7 (105.5–136.6)	153.2 (127.8–185.0)	< 0.001
Mean bedside blood glucose (mg/dL)	133.7 (115.8–159.9)	124.7 (110.1–142.0)	157.3 (134.8–184.5)	< 0.001
Minimum blood glucose (mg/dL)	87.0 (74.0–100.0)	87.0 (76.0–99.0)	85.0 (67.0–104.0)	< 0.001
Severe hypoglycemia (Minimum blood glucose ≤40 mg/dL)	1410 (2.7)	668 (1.8)	742 (4.7)	< 0.001
Moderate hypoglycemia (Minimum blood glucose 41-70 mg/dL)	9178 (17.6)	5293 (14.5)	3885 (24.8)	< 0.001
Standard of deviation	26.3 (17.3–40.8)	22.0 (15.0–31.8)	42.0 (28.8–59.5)	< 0.001
Coefficient of variation (%)	20.5 (14.7–28.1)	18.4 (13.2–24.6)	26.6 (19.9–34.7)	< 0.001
Number of BG measurements	21.0 (8.0–45.0)	15.0 (6.0–36.0)	36.0 (19.0–64.0)	< 0.001
ICU mortality	3792 (7.3)	2568 (7.0)	1224 (7.8)	0.002
Hospital mortality	6494 (12.5)	4398 (12.1)	2096 (13.4)	< 0.001
ICU LOS (days)	3.7 (2.7–5.9)	3.7 (2.7–5.9)	3.7 (2.7–5.9)	0.442
Hospital LOS (days)	8.2 (5.3–13.4)	8.1 (5.2–13.4)	8.4 (5.5–13.5)	< 0.001

*APACHE* Acute Physiology and Chronic Health Evaluation, *BMI* Body mass index, *ICU* Intensive care unit, *LOS* Length of stay, *TWA* Time-weighted average

Number shown as n (%) or median (IQR) as appropriate

^a^ Percentage do not add up to 100% because of missing data

^b^ Time weighted average blood glucose was calculated using both laboratory measurement and bedside testing

Compared with nondiabetic patients, patients with diabetes had higher TWA glucose and mean blood glucose levels. The occurrences of severe hypoglycemia (≤40 mg/dL) and moderate hypoglycemia (41–70 mg/dL) were higher in diabetic patients. They had a higher standard deviation and CV of the glucose level.

Table 2 compares the patient demographics and glycemic control for hospital survivors and nonsurvivor, stratified by diabetic status. Compared with survivors, non-survivors had higher TWA glucose among both nondiabetic and diabetic patients. Hypoglycemia was more commonly found in nonsurvivors. The CV was higher in nonsurvivors among both nondiabetes and diabetes patients.

**Table 2:** Patient demographics, glycemic control of hospital survivors and nonsurvivors, stratified by diabetic status

	No diabetes			Diabetes		
	Hospital survivors (*n* = 32,057)	Hospital nonsurvivors (*n* = 4398)	*p*-value	Hospital survivors (*n* = 13,556)	Hospital nonsurvivors (*n* = 2096)	*p*-value
Age (years) ^a^	65.0 (52.0–77.0)	70.0 (58.0–81.0)	< 0.001	68.0 (59.0–77.0)	71.0 (62.0–79.0)	< 0.001
Male ^a^	17,561 (54.8)	2388 (54.3)	0.557	7423 (54.8)	1141 (54.4)	0.802
BMI (kg/m^2^) ^a^						
< 18.5	3235 (10.1)	605 (13.8)	< 0.001	615 (4.5)	137 (6.5)	< 0.001
18.5- < 25	8858 (27.6)	1290 (29.3)		2608 (19.2)	455 (21.7)	
25- < 30	7305 (22.8)	989 (22.5)		2853 (21.0)	436 (20.8)	
30- < 35	5218 (16.3)	635 (14.4)		2507 (18.5)	402 (19.2)	
35- < 40	3011 (9.4)	341 (7.8)		1786 (13.2)	254 (12.1)	
≥ 40	3381 (10.5)	411 (9.3)		2750 (20.3)	358 (17.1)	
APACHE IV ^a^	55.0 (41.0–72.0)	81.0 (62.0–104.0)	< 0.001	60.0 (47.0–77.0)	82.0 (65.0–105.0)	< 0.001
Admission diagnosis ^a^						
Medical	24,738 (77.2)	3917 (89.1)	< 0.001	10,810 (79.7)	1922 (91.7)	< 0.001
Surgical	6927 (21.6)	467 (10.6)		2726 (20.1)	174 (8.3)	
Elective	5911 (18.4)	342 (7.8)		2437 (18.0)	123 (5.9)	
Emergency	1016 (3.2)	125 (2.8)		289 (2.1)	51 (2.4)	
Mechanical ventilation ^a^	10,578 (33.0)	2382 (54.1)	< 0.001	4610 (34.0)	1216 (58.0)	< 0.001
On inotropes or vasopressors	6519 (20.3)	1970 (44.8)	< 0.001	3095 (22.8)	925 (44.1)	< 0.001
Trauma	2034 (6.3)	195 (4.4)	< 0.001	303 (2.2)	44 (2.1)	0.754
TWA blood glucose (mg/dL)^b^	116.5 (104.6–131.9)	131.8 (115.7–152.0)	< 0.001	157.0 (132.1–185.5)	164.9 (141.6–192.0)	< 0.001
Mean blood glucose (mg/dL)	118.5 (106.0–133.7)	132.7 (116.4–153.2)	< 0.001	155.3 (131.8–182.7)	164.5 (153.2–190.4)	< 0.001
Mean laboratory blood glucose (mg/dL)	117.0 (104.6–133.5)	135.3 (117.1–156.9)	< 0.001	151.0 (126.1–182.0)	170.0 (141.7–200.1)	< 0.001
Mean bedside blood glucose (mg/dL)	123.6 (109.4–140.0)	133.1 (115.8–154.1)	< 0.001	156.6 (134.0–184.1)	161.4 (140.3–187.8)	< 0.001
Minimum blood glucose (mg/dL)	87.0 (77.0–98.0)	86.0 (69.0–103.0)	< 0.001	85.0 (67.0–104.0)	83.0 (61.0–108.0)	0.108
Severe hypoglycemia (Minimum blood glucose ≤40 mg/dL)	415 (1.3)	253 (5.8)	< 0.001	574 (4.2)	168 (8.0)	< 0.001
Moderate hypoglycemia (Minimum blood glucose 41-70 mg/dL)	4390 (13.7)	903 (20.5)	< 0.001	3337 (24.6)	548 (26.1)	0.139
Standard of deviation	21.3 (14.5–30.5)	29.2 (20.2–41.7)	< 0.001	41.1 (28.3–58.8)	46.3 (32.8–64.2)	< 0.001
Coefficient of variation (%)	17.9 (12.9–23.9)	22.0 (15.9–30.0)	< 0.001	26.3 (19.7–34.3)	28.8 (21.4–36.9)	< 0.001
Number of BG measurements	14.0 (6.0–34.0)	21.0 (9.0–45.0)	< 0.001	36.0 (19.0–62.0)	37.0 (19.0–72.0)	< 0.001

*APACHE* Acute Physiology and Chronic Health Evaluation, *BMI* Body mass index, *ICU* Intensive care unit, *LOS* Length of stay, *TWA* Time weighted average

Number shown as n (%) or median (IQR) as appropriate

^a^ Percentage do not add up to 100% because of missing data

^b^ Time weighted average blood glucose was calculated using both laboratory measurement and bedside testing

### Relationship between glycemic control and mortality

**Fig. 1:**
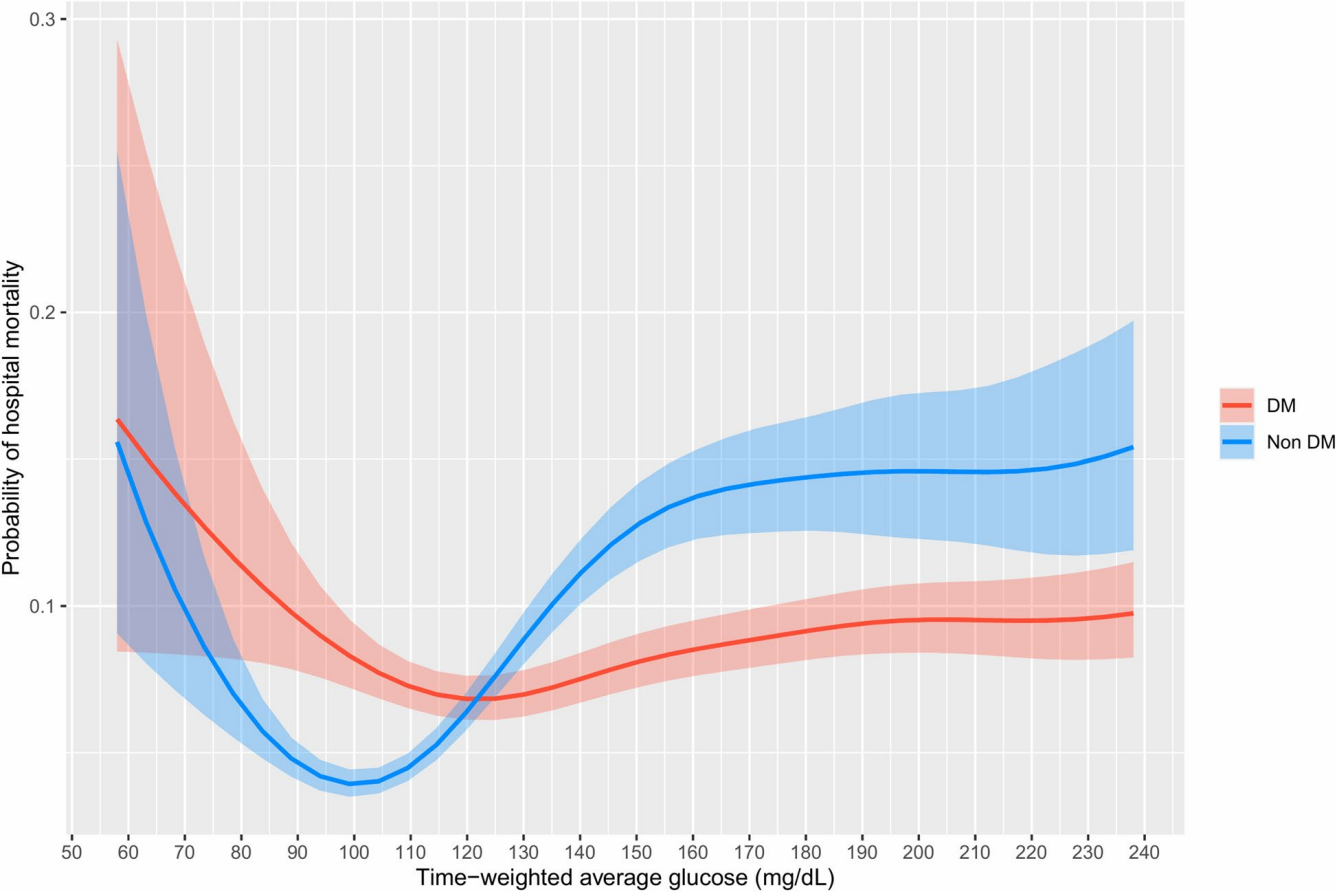
Probability of hospital mortality and time-weighted average glucose in diabetes and nondiabetes patients. DM, diabetes mellitus. Analysis was adjusted for age, APACHE IV scores, body mass index, admission diagnosis, mechanical ventilation, and use of vasopressor or inotropic agents

Figure 1 shows the J-curved association between the probability of hospital mortality and TWA glucose levels in diabetes and nondiabetes patients, adjusted for age, APACHE IV scores, BMI, admission diagnosis, mechanical ventilation, and the use of vasopressor or inotropic agents. Supplementary Table 4 shows the OR comparing specific TWA glucose levels and demonstrates the attenuated response to hyperglycemia in diabetic patients. Using TWA glucose of 100 mg/dL as the reference value, the adjusted OR for TWA glucose of 140 mg/dL in patients with no diabetes and patients with diabetes were 3.05 (3.03–3.08) and 1.14 (1.08–1.20) respectively. Using the same reference value, the adjusted OR for TWA glucose of 180 mg/dL was 4.20 (4.07–4.33) in patients with no diabetes, and 1.49 (1.41–1.57) in patients with diabetes; the adjusted OR for TWA of 80 mg/dL was 1.74 (1.57–1.92) in patients with no diabetes, and 1.36 (1.12–1.66) in patients with diabetes. The glucose range with below-average risk of hospital mortality was identified from the individual association curves for nondiabetic and diabetic patients (Supplementary Figure 2). Patients with no history of diabetes had a below-average risk of mortality with a glycemic range of approximately 80–120 mg/dL. There was a steep rise in mortality seen with both hyperglycemia and hypoglycemia outside this range. Conversely, the association between mortality and TWA glucose was more blunted in diabetes patients than in nondiabetes patients. The below-average risk was right-shifted to the range of 90–150 mg/dL. In particular, the risk of mortality with hyperglycemia was attenuated in diabetic patients.

**Fig. 2:**
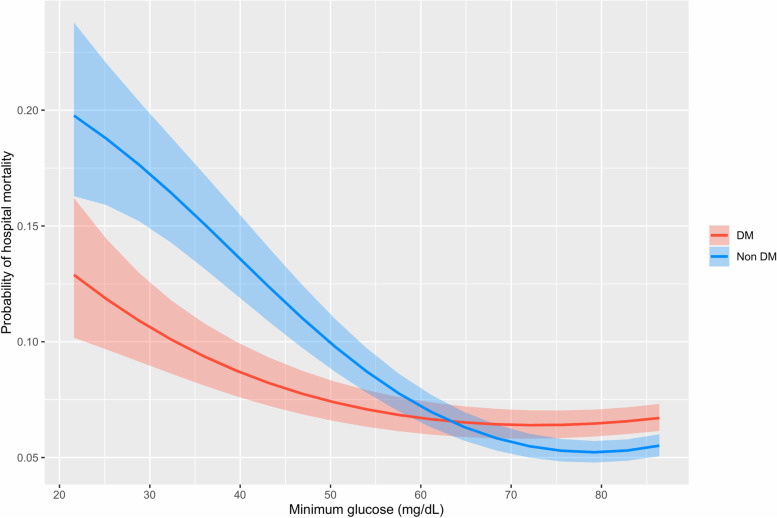
Probability of hospital mortality and minimum glucose in diabetes and nondiabeties patients. DM, diabetes mellitus. Analysis was adjusted for age, APACHE IV scores, body mass index, admission diagnosis, mechanical ventilation, and use of vasopressor or inotropic agents

Figure 2 illustrates the association between the probability of hospital mortality and hypoglycemia. There was an almost linear relationship between the severity of hypoglycemia and hospital mortality. A lower minimum blood glucose was associated with increased hospital mortality, but the effect was less pronounced in diabetes patients (Supplementary Table 4).

**Fig. 3:**
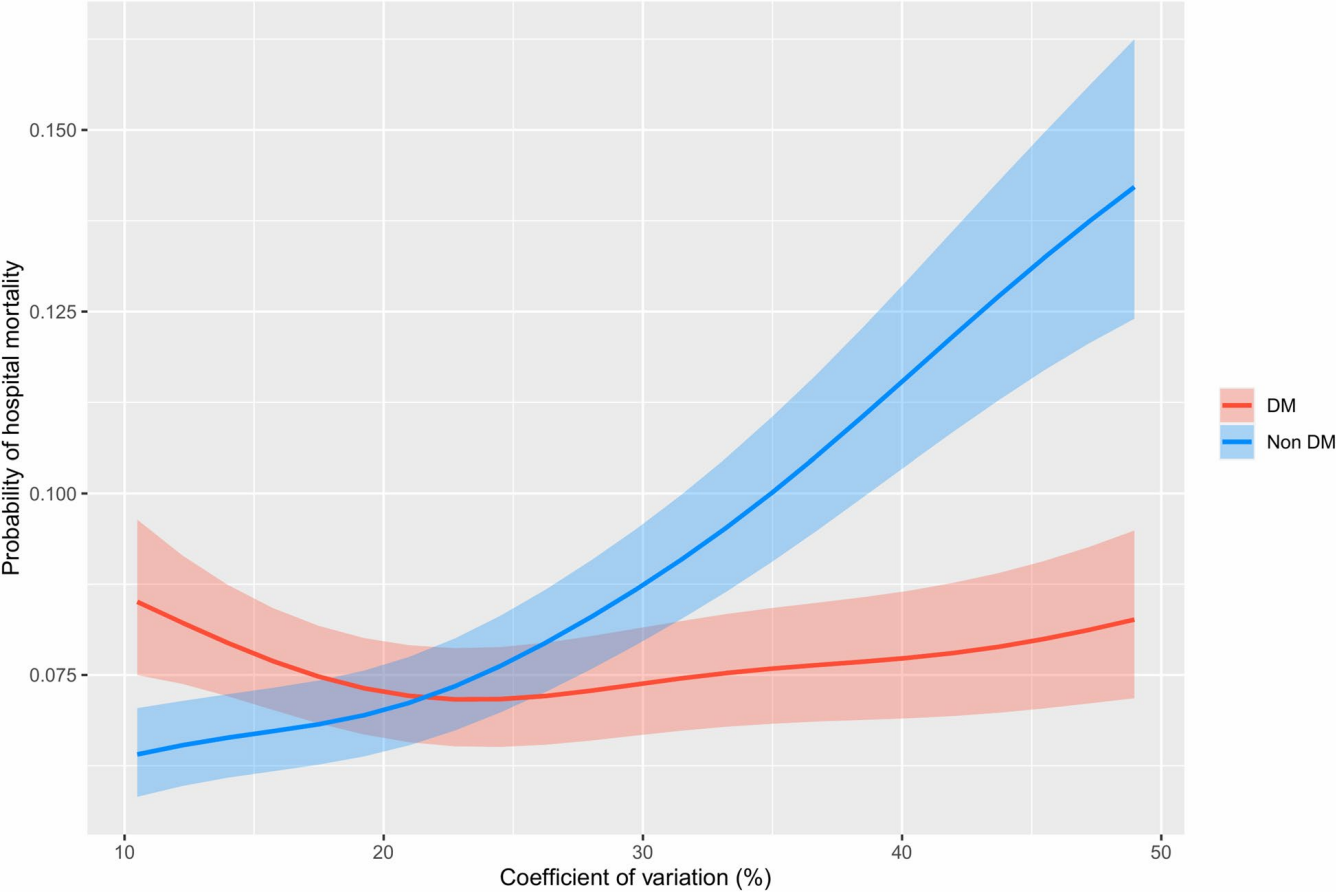
Probability of hospital mortality and coefficient of variation in diabetes and nondiabetes patients. DM, diabetes mellitus. Analysis was adjusted for age, APACHE IV scores, body mass index, admission diagnosis, mechanical ventilation, and use of vasopressor or inotropic agents

Figure 3 shows the relationship between the CV and hospital mortality. CV was positively associated with increased mortality in nondiabetic patients, but this relationship was not demonstrated in patients with diabetes in the adjusted analysis (Supplementary Table 4).

### Subgroup analysis

After adjusting for diabetic status, age, APACHE IV scores, BMI, mechanical ventilation, and use of vasopressor or inotropic agents, for patients admitted for surgical diagnoses, the association curve between TWA glucose and hospital mortality was more flattened than that of patients admitted for medical diagnoses (Supplementary Figure 3). The association between hypoglycemia and mortality was less pronounced in surgical patients (Supplementary Figure 4). An increase in glucose variability was associated with increased mortality in both groups of patients (Supplementary Figure 5).

The associations between TWA glucose and mortality were similar between trauma and non-trauma patients (Supplementary Figure 6). The response to hypoglycemia and glucose variability were also similar between the two groups (Supplementary Figures 7 and 8).

### Sensitivity analysis

In patients with IDDM and NIDDM on OHA, a flattened curve of association between TWA glucose and mortality was observed compared with that for the nondiabetic patients. The positive association between hyperglycemia and mortality in patients with NIDDM on diet was between that of nondiabeties and IDDM patients (Supplementary Figure 9). There was an overlapping response to hypoglycemia in patients with no diabetes and patients with diabetes (Supplementary Figure 10). The positive association between glucose variability and mortality was observed in nondiabetic patients and, to a lesser degree, in NIDDM patients. The relationship between glucose variability and mortality appeared to be blunted in IDDM patients (Supplementary Figure 11).

Sensitivity analysis of all patients including those who stayed in the ICU for < 2 days, showed a similar J-shaped curve in nondiabetic patients and a similar flattened curve in diabetic patients. However, the risk of mortality with glucose levels ranging below 70 mg/dL was higher in both groups than in patients with LOS ≥ 2 days (Supplementary Figure 12). Comparing with patients with LOS ≥ 2 days, the response to hypoglycemia and glucose variability was similar (Supplementary Figures 13 and 14).

Supplementary Table 5 shows the results of the Cox proportional hazard model with glucose as the time-varying covariate on ICU mortality. After adjusting for diabetic status, age, APACHE IV scores, BMI, mechanical ventilation, and use of vasopressor or inotropic agents, the hazard ratio of glucose was 1.003 (95% CI 1.002–1.004, *p*-value < 0.001). The Schoenfeld global and individual *p*-values for glucose were < 0.001 and 0.007 respectively. The smoothed scaled Schoenfeld residual plot of glucose showed that the impact of glucose on ICU mortality was relatively constant during the ICU stay (Supplementary Table 6 and Supplementary Figure 15).

## Discussion

### Summary of findings

This retrospective analysis of 52,107 patients from the eICU database revealed disparities in the relationships between glycemic control and hospital mortality in diabetic and nondiabetic patients. Patients with no diabetes demonstrated a J-shaped association of TWA glucose and mortality, with a below average risk of mortality in the range of 80–120 mg/dL. In contrast, diabetic patients showed a right-shifted and attenuated association between TWA glucose and hospital mortality, with a below-average risk of mortality ranging from 90 to 150 mg/dL. Hypoglycemia was associated with increased mortality in both groups but to a lesser extent in diabetic patients. An association was observed between the CV and hospital mortality in nondiabetic patients only. In the subgroup analysis, surgical and trauma patients appeared to better tolerate lower ranges of glucose. Sensitivity analysis confirmed that patients on insulin or oral hypoglycemic agents had a more blunted response to hyperglycemia, and hypoglycemia, and were less affected by glucose variability. The change in the effect of glucose on survival over time was small and the statistically significant Schoenfeld test was more likely related to the large sample size [23].

### Comparison with previous studies

The relationship between hyperglycemia and mortality in the current study offered evidence against leaving hyperglycemia uncontrolled. While the early randomized controlled trials including Leuven I and Leuven II supported tight glucose control [3, 29], it has fallen out of favor since the NICE-SUGAR study, which advocated a single glucose range of 140–180 mg/dL [4]. Accurate glucose measurements using a blood gas analyzer instead of various inaccurate glucometers, the measurement of reliable arterial blood samples instead of venous blood or capillary samples, frequent blood glucose measurements, standardized insulin protocols and an experienced nursing team all contribute to a safe and effective glucose control. These factors were questioned in the NICE-SUGAR study and this disparity was argued to explain the conflicting evidence from the Leuven studies and NICE-SUGAR [18, 30–32]. In our study, a TWA glucose range of 80–120 mg/dL was associated with below-average risk of mortality in patients with no history of diabetes. It was much lower than the widely adopted target of 140–180 mg/dL from the NICE-SUGAR study.

Furthermore, the glucose target from the NICE-SUGAR study did not account for the diabetic status [4], and this warrants significant attention because the prevalence of diabetes has been increasing over the years globally [33], which is also reflected by the increasing proportion of diabetic patients across studies (30% in the current cohort, 20% in NICE-SUGAR, 17% in Leuven II and 13% in Leuven I). Benefits from glucose control might be less marked in diabetic patients because of their adaptive modulation of glucose transporters due to chronic hyperglycemia. In our study, patients on insulin had a lower risk of mortality to hyperglycemia than patients on OHA or diet. This might be explained by a new “set point” of hyperglycemia caused by the cellular downregulation of GLUT4 transporters induced by exogenous insulin [34–36]. This also echoed the findings by Liao et al. which demonstrated that the effect of admission glycemia on mortality had to be assessed with reference to the preadmission glycemia [37]. In our study, a TWA glucose range of 90–150 mg/dL was associated with below-average risk of mortality in patients with diabetes. It was also lower than the target from the NICE-SUGAR study. The difference in relationships between TWA glucose and mortality in patients with or without diabetes supported the use of individualised glucose targets based on diabetic status. While the CONTROLING study did not demonstrate a mortality benefit towards individualised blood glucose targets [12], the narrow difference in time-weighted mean blood glucose and the insufficient power were its major limitations [38].

### Implications of findings and future directions

Existing glucose targets of either 80–110 mg/dL or 140–180 mg/dL in diabetic and nondiabetic patients would be too low for one group and too high for the other. At the same time, leaving the glucose level too high to avoid hypoglycemia is ‘throwing out the baby with the bathwater’ [32]. Since the main barrier to tight glucose control is the risk of hypoglycemia, the direction should be changed to introduce measures to prevent hypoglycemia while maintaining glucose in the optimal range. These approaches include accurate measurement tools such as blood gas analyzers instead of home-use glucometers and highly experienced nursing care with frequent monitoring and adherence to validated insulin protocols [32]. The use of continuous glucose monitoring (CGM) may reduce hypoglycemic episodes [39] and decrease the nursing workload [40]. However, CGM would not improve clinical outcomes unless it is accompanied by CGM-specific insulin protocols [41]. ‘Closed-loop’ systems are feasible [42, 43] and should be considered as the future direction in glycemic control, although the insulin regimen would require further validation for critically-ill patients.

The small effect size of glycemic control on mortality would mandate an enormous sample size to produce a meaningful clinical effect [8, 31], not to mention patients with different characteristics, such as those with diabetes, or patients with different diagnoses. Spending resources and time on arbitrarily defined glucose targets is unwise and may be harmful to patients. The analysis of existing large ICU databases together with GAMs allowed the modeling of complex biological relationships. The increasing volume of data from electronic health records would be the catalyst for big data analysis and could yield valuable insights for future clinical trials, such as mean arterial pressure and oxygen saturation targets [44, 45].

### Strengths and limitations

Data from the eICU dataset were collected within 2 years and well after the NICE-SUGAR trial. Therefore, the glycemic control strategy and other ICU management practices may be more uniform than those in previous observational studies using data spreading across 10 years [46]. The use of GAMs allowed visualization of the relationship between glycemic control and mortality and identification of the below-average risk glucose range.

Several limitations existed in our study. First, HbA1c levels were not available from the database. The diabetic population is not homogenous and patients on the same diabetic medications could have very different levels of glucose control. Without HbA1c data, patients with undiagnosed diabetes would be classified as nondiabetic. Moreover, the relationship between glycemic control and mortality was confounded by preadmission glycemia, which might be determined using HbA1c [6, 37]. We attempted to perform a sensitivity analysis according to the use of diabetic medications to reflect the stages of diabetes. Second, information on methods of glucose measurement and types of blood sampling were not available from the dataset. Data on the dosage of total parenteral nutrition and the use of corticosteroids before admission were also incomplete. Third, while TWA glucose takes time into consideration, it suffers from the variability in the frequency of glucose measurements. Patients with more glucose measurements would likely have a better representation of their glucose variation. Lastly, this retrospective study can only suggest associations but not causation. It remains unclear whether glucose alterations are causally related to outcome, or innocent bystander reflecting underlying disease severity. While this study might not fully solve the puzzle of optimal glucose targets, it suggested that future research is needed to develop a model with optimal glucose targets considering HbA1c and to evaluate the clinical outcome of individualized glucose targets.

## Conclusions

Time-weighted average glucose, hypoglycemia, and glucose variability had different impacts on clinical outcomes in patients with and without diabetes. Compared with nondiabetic patients, diabetic patients showed a more blunted response to hypo- and hyperglycemia and glucose variability. Glycemic control strategies should be reconsidered to avoid both hypoglycemia and hyperglycemia.

## Supplementary Information

**Additional file 1: Supplementary Table 1.** Measures of glycemic control**. Supplementary Table 2.** Number of nondiabetes and diabetes patients on insulin, oral hypoglycemic agents, and diet. **Supplementary Table 3.** Top ten admission diagnoses in patients admitted for medical or surgical diagnoses. **Supplementary Table 4.** Odds ratios for the association of hospital mortality on glycemic measures derived from generalised additive model. **Supplementary Table 5.** Hazard ratios from the Cox proportional hazard model with glucose as time-varying covariate on ICU mortality. **Supplementary Table 6.** Schoenfeld’s global and individual test for the violation of proportional assumptions of Cox proportional hazard model. **Supplementary Figure 1.** Patient flow chart. **Supplementary Figure 2.** Graphical representation of the generalised additive model showing the time weighted average glucose associated with below-average risk of mortality for a) patients with no diabetes and b) patients with diabetes. **Supplementary Figure 3.** Probability of hospital mortality and time weighted average glucose in medical and surgical patients. **Supplementary Figure 4.** Probability of hospital mortality and minimum glucose in medical and surgical patients. **Supplementary Figure 5.** Probability of hospital mortality and coefficient of variation in medical and surgical patients. **Supplementary Figure 6.** Probability of hospital mortality and time weighted average glucose in trauma and nontrauma patients. **Supplementary Figure 7.** Probability of hospital mortality and minimum glucose in trauma and nontrauma patients. **Supplementary Figure 8.** Probability of hospital mortality and coefficient of variation in trauma and nontrauma patients. **Supplementary Figure 9.** Probability of hospital mortality and time weighted average glucose in diabetes patients on insulin, oral hypoglycemic agents, or diet and patients with no diabetes. **Supplementary Figure 10.** Probability of hospital mortality and minimum glucose in diabetes patients on insulin, oral hypoglycemic agents, or diet and patients with no diabetes. **Supplementary Figure 11.** Probability of hospital mortality and coefficient of variation in diabetes patients on insulin, oral hypoglycemic agents, or diet and patients with no diabetes. **Supplementary Figure 12.** Probability of hospital mortality and time weighted average glucose in all diabetes and nondiabetes patients (including length of stay < 2 days). **Supplementary Figure 13.** Probability of hospital mortality and minimum glucose in all diabetes and nondiabetes patients (including length of stay < 2 days). **Supplementary Figure 14.** Probability of hospital mortality and coefficient of variation in all diabetes and nondiabetes patients (including length of stay < 2 days). **Supplementary Figure 15.** Smoothed scaled Schoenfeld residual plot of glucose on ICU mortality.

**Additional file 2.** STROBE Statement.

## Data Availability

All data generated or analyzed during the present study are included in this published article and its supplementary information files.
