# Supplementary material for: Glycemic control in critically ill patients with or without diabetes
Source: BMC Anesthesiol. 2022 Jul 16;22:227. doi: 10.1186/s12871-022-01769-4 (PMC9288031; doi:10.1186/s12871-022-01769-4)

**Glycemic control in critically ill patients with or without diabetes**

Supplementary Information

[Supplementary table 1. Measures of glycemic control 2](#_Toc106059656)

[Supplementary table 2. Number of nondiabetes and diabetes patients on insulin, oral hypoglycemic agents, and diet 3](#_Toc106059657)

[Supplementary table 3. Top ten admission diagnoses in patients admitted for medical or surgical diagnoses 4](#_Toc106059658)

[Supplementary table 4. Odds ratios for the association of hospital mortality on glycemic measures derived from generalised additive model 5](#_Toc106059659)

[Supplementary table 5. Hazard ratios from the Cox proportional hazard model with glucose as time-varying covariate on ICU mortality 6](#_Toc106059660)

[Supplementary table 6. Schoenfeld's global and individual test for the violation of proportional assumptions of Cox proportional hazard model 7](#_Toc106059661)

[Supplementary figure 1. Patient flow chart 8](#_Toc106060532)

[Supplementary figure 2. Graphical representation of the generalised additive model showing the time weighted average glucose associated with below-average risk of mortality for a) patients with no diabetes and b) patients with diabetes 9](#_Toc106060533)

[Supplementary figure 3. Probability of hospital mortality and time weighted average glucose in medical and surgical patients 11](#_Toc106060534)

[Supplementary figure 4. Probability of hospital mortality and minimum glucose in medical and surgical patients 12](#_Toc106060535)

[Supplementary figure 5. Probability of hospital mortality and coefficient of variation in medical and surgical patients 13](#_Toc106060536)

[Supplementary figure 6. Probability of hospital mortality and time weighted average glucose in trauma and nontrauma patients 14](#_Toc106060537)

[Supplementary figure 7. Probability of hospital mortality and minimum glucose in trauma and nontrauma patients 15](#_Toc106060538)

[Supplementary figure 8. Probability of hospital mortality and coefficient of variation in trauma and nontrauma patients 16](#_Toc106060539)

[Supplementary figure 9. Probability of hospital mortality and time weighted average glucose in diabetes patients on insulin, oral hypoglycemic agents, or diet and patients with no diabetes 17](#_Toc106060540)

[Supplementary figure 10. Probability of hospital mortality and minimum glucose in diabetes patients on insulin, oral hypoglycemic agents, or diet and patients with no diabetes 18](#_Toc106060541)

[Supplementary figure 11. Probability of hospital mortality and coefficient of variation in diabetes patients on insulin, oral hypoglycemic agents, or diet and patients with no diabetes 19](#_Toc106060542)

[Supplementary figure 12. Probability of hospital mortality and time weighted average glucose in all diabetes and nondiabetes patients (including length of stay < 2 days) 20](#_Toc106060543)

[Supplementary figure 13. Probability of hospital mortality and minimum glucose in all diabetes and nondiabetes patients (including length of stay < 2 days) 21](#_Toc106060544)

[Supplementary figure 14. Probability of hospital mortality and coefficient of variation in all diabetes and nondiabetes patients (including length of stay < 2 days) 22](#_Toc106060545)

[Supplementary figure 15. Smoothed scaled Schoenfeld residual plot of glucose on ICU mortality 23](#_Toc106060546)

Supplementary table 1. Measures of glycemic control

| Measures | Details |
| --- | --- |
| Time weighted average (TWA)  glucose | The area under the curve (AUC) was first calculated  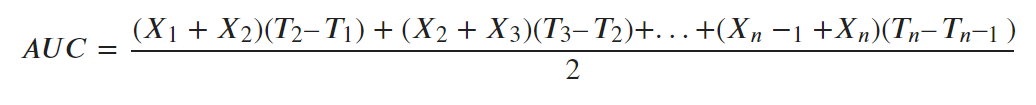  where X_n_ is the glucose value, X_1_ is the first glucose value after ICU admission, X_n_ is the last value in the ICU admission, and T_n_ is the time the glucose value is taken  then the AUC was divided by the time (Tn-T1)  then:  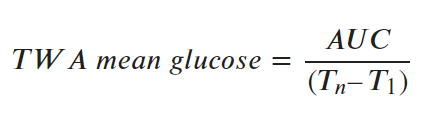 |
| Coefficient of variation (CV) | 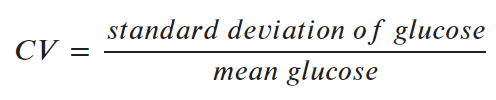 |

Supplementary table 2. Number of nondiabetes and diabetes patients on insulin, oral hypoglycemic agents, and diet

| Patients | n |
| --- | --- |
| Insulin-dependent diabetes | 6454 |
| Non-insulin-dependent diabetes on oral hypoglycemic agents | 6366 |
| Non-insulin-dependent diabetes on diet | 1658 |
| Diabetes (not known whether on insulin/ oral hypoglycemic agents/ diet) | 1174 |
| Patients with no diabetes | 36455 |

Supplementary table 3. Top ten admission diagnoses in patients admitted for medical or surgical diagnoses

| Medical | | Surgical | |
| --- | --- | --- | --- |
| Diagnosis | n | Diagnosis | n |
| Sepsis, pulmonary | 3498 | CABG alone, coronary artery bypass grafting | 1662 |
| Congestive heart failure | 2031 | Aortic valve replacement (isolated) | 793 |
| Cerebrovascular accident/stroke | 1972 | GI obstruction, surgery for (including lysis of adhesions) | 390 |
| Cardiac arrest (with or without respiratory arrest) | 1946 | GI perforation/rupture, surgery for | 375 |
| Sepsis, renal/UTI (including bladder) | 1861 | CABG with aortic valve replacement | 323 |
| Infarction, acute myocardial | 1593 | Mitral valve replacement | 215 |
| Pneumonia, bacterial | 1520 | Thoracotomy for lung cancer | 191 |
| Emphysema/bronchitis | 1308 | Neoplasm-cranial, surgery for (excluding transphenoidal) | 174 |
| Rhythm disturbance (atrial, supraventricular) | 1092 | Hematoma subdural, surgery for | 170 |
| Arrest, respiratory (without cardiac arrest) | 1024 | Cancer-colon/rectal, surgery for (including abdominoperineal resections) | 158 |

CABG, coronary artery bypass graft; GI, gastrointestinal; UTI, urinary tract infection

Supplementary table 4. Odds ratios for the association of hospital mortality on glycemic measures derived from generalised additive model

|  | Patients with no diabetes | | Patients with diabetes | |
| --- | --- | --- | --- | --- |
|  | Crude OR (95% CI) | Adjusted OR (95% CI) | Crude OR (95% CI) | Adjusted OR (95% CI) |
| TWA glucose (mg/dL) | | | | |
| 60 vs. 100 | 4.66 (2.78 – 7.80) | 4.34 (2.69 – 7.01) | 2.17 (1.17 – 4.03) | 1.91 (1.14 – 3.19) |
| 80 vs. 100 | 1.78 (1.57 – 2.02) | 1.74 (1.57 – 1.92) | 1.45 (1.12 – 1.88) | 1.36 (1.12 – 1.66) |
| 120 vs. 100 | 1.84 (1.80 – 1.89) | 1.66 (1.64 – 1.67) | 0.88 (0.83 – 0.94) | 0.91 (0.87 – 0.95) |
| 140 vs. 100 | 3.85 (3.78 – 3.92) | 3.05 (3.03 – 3.08) | 1.21 (1.11 – 1.32) | 1.14 (1.08 – 1.20) |
| 160 vs. 100 | 5.39 (5.37 – 5.41) | 3.84 (3.81 – 3.87) | 1.55 (1.41 – 1.70) | 1.37 (1.30 – 1.45) |
| 180 vs. 100 | 6.03 (5.84 – 6.22) | 4.20 (4.07 – 4.33) | 1.67 (1.53 – 1.83) | 1.49 (1.41 – 1.57) |
| 180 vs. 120 | 3.27 (3.09 – 3.46) | 2.53 (2.43 – 2.64) | 1.89 (1.84 – 1.94) | 1.65 (1.63 – 1.66) |
| 180 vs. 150 | 1.26 (1.21 – 1.32) | 1.18 (1.22 – 1.14) | 1.17 (1.17 – 1.18) | 1.16 (1.16 – 1.17) |
| Minimum glucose (mg/dL) | | | | |
| 70 vs. 80 | 1.37 (1.36 – 1.37) | 1.11 (1.11 – 1.12) | 1.08 (1.07 – 1.08) | 1.00 (1.00 – 1.00) |
| 60 vs. 80 | 2.16 (2.12 – 2.21) | 1.47 (1.44 – 1.50) | 1.23 (1.22 – 1.24) | 1.07 (1.06 – 1.07) |
| 50 vs. 80 | 3.39 (3.26 – 3.52) | 2.03 (1.96 – 2.10) | 1.48 (1.46 – 1.51) | 1.21 (1.19 – 1.22) |
| 40 vs. 80 | 4.82 (4.49 – 5.18) | 2.72 (2.56 – 2.89) | 1.86 (1.78 – 1.94) | 1.45 (1.42 – 1.48) |
| 30 vs. 80 | 6.43 (5.82 – 7.10) | 3.53 (3.24 – 3.85) | 2.42 (2.23 – 2.64) | 1.82 (1.73 – 1.91) |
| Coefficient of variation (%) | | | | |
| 20 vs. 10 | 1.66 (1.61 – 1.70) | 1.11 (1.10 – 1.12) | 1.18 (1.14 – 1.22) | 0.91 (0.88 – 0.95) |
| 30 vs. 10 | 2.63 (2.60 – 2.66) | 1.41 (1.41 – 4.42) | 1.40 (1.33 – 1.46) | 0.93 (0.89 – 0.96) |
| 40 vs. 10 | 4.14 (4.07 – 4.22) | 1.92 (1.89 – 1.96) | 1.65 (1.60 – 1.70) | 0.98 (0.94 – 1.01) |
| 50 vs. 10 | 5.78 (5.43 – 6.16) | 2.50 (2.37 – 2.65) | 1.95 (1.95 – 1.95) | 1.05 (1.04 – 1.06) |

CI, confidence interval; OR, Odds ratio; TWA, time weighted average

Supplementary table 5. Hazard ratios from the Cox proportional hazard model with glucose as time-varying covariate on ICU mortality

| Covariate | Coefficient(b_i_) | HR [exp(b_i_)] (95% CI) | p-value |
| --- | --- | --- | --- |
| Glucose | 0.003 | 1.003 (1.002 – 1.004) | <0.001 |
| Diabetes | -0.143 | 0.867 (0.799 – 0.940) | <0.001 |
| Age (years) | 0.012 | 1.012 (1.009 – 1.014) | <0.001 |
| APACHE IV | 0.017 | 1.017 (1.016 – 1.019) | <0.001 |
| Admission diagnosis |  |  |  |
| Operative | -0.694 | 0.500 (0.446 – 0.560) | <0.001 |
| BMI (kg/m^2^) |  |  |  |
| < 18.5 | Reference | - | - |
| 18.5-<25 | -0.172 | 0.842 (0.744 – 0.952) | 0.006 |
| 25-<30 | -0.312 | 0.732 (0.645 – 0.831) | <0.001 |
| 30-<35 | -0.298 | 0.743 (0.650 – 0.849) | <0.001 |
| 35-<40 | -0.185 | 0.831 (0.717 – 0.963) | 0.014 |
| ≥40 | -0.329 | 0.719 (0.624 – 0.829) | <0.001 |
| Mechanical ventilation | 0.163 | 1.177 (1.091 – 1.271) | <0.001 |
| On inotropes or vasopressors | 0.501 | 1.650 (1.532 – 1.778) | <0.001 |

APACHE, Acute Physiology and Chronic Health Evaluation; BMI, Body mass index; CI, confidence interval; HR, Hazard ratios;

Supplementary table 6. Schoenfeld's global and individual test for the violation of proportional assumptions of Cox proportional hazard model

| Covariate | p-value |
| --- | --- |
| Glucose | 0.007 |
| Diabetes | 0.045 |
| Age | <0.001 |
| APACHE IV | <0.001 |
| Admission diagnosis | <0.001 |
| BMI | 0.752 |
| Mechanical ventilation | <0.001 |
| On inotropes or vasopressors | <0.001 |
| Global | <0.001 |

Supplementary figure 1. Patient flow chart


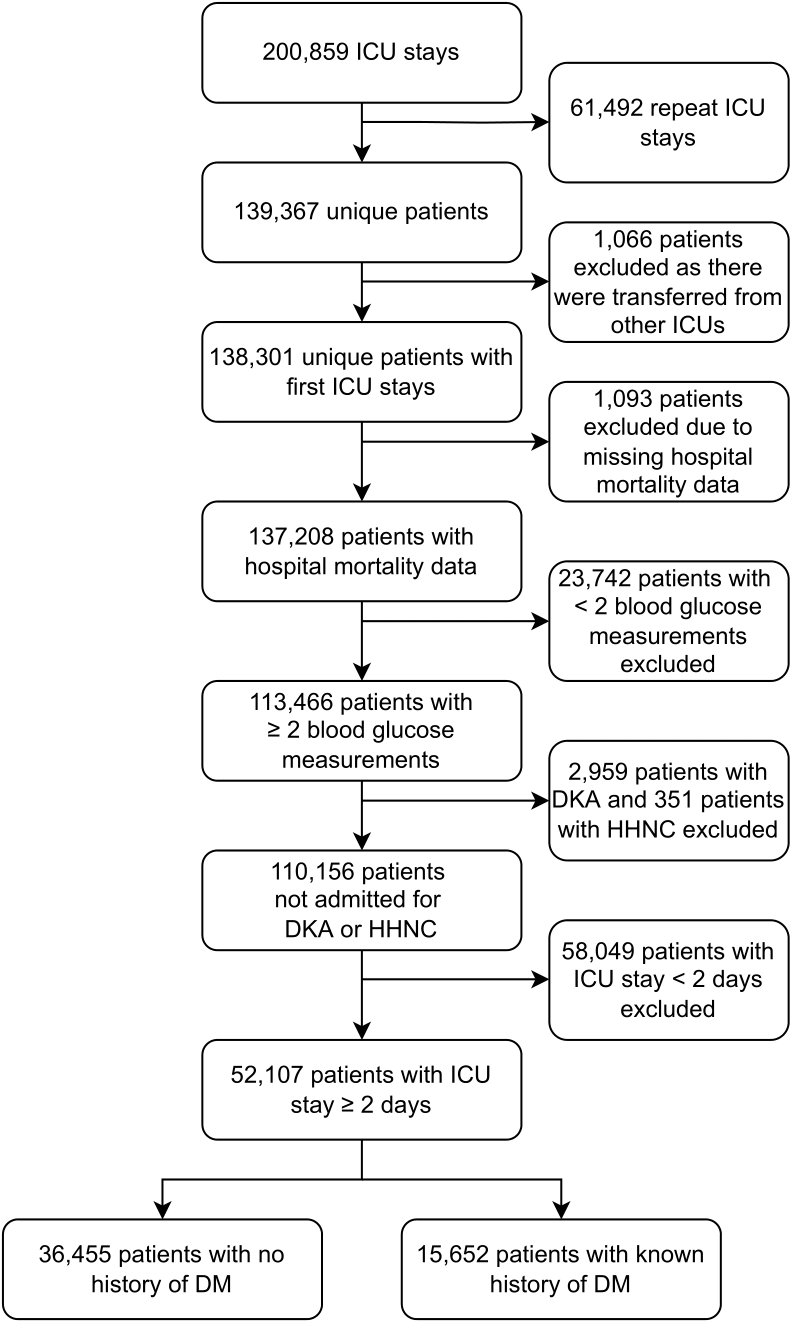


DM, diabetes mellitus; DKA, diabetic ketoacidosis; HHNC, diabetic hyperglycemic hyperosmolar nonketotic coma; ICU, intensive care unit

Supplementary figure 2. Graphical representation of the generalised additive model showing the time weighted average glucose associated with below-average risk of mortality for a) patients with no diabetes and b) patients with diabetes

a)


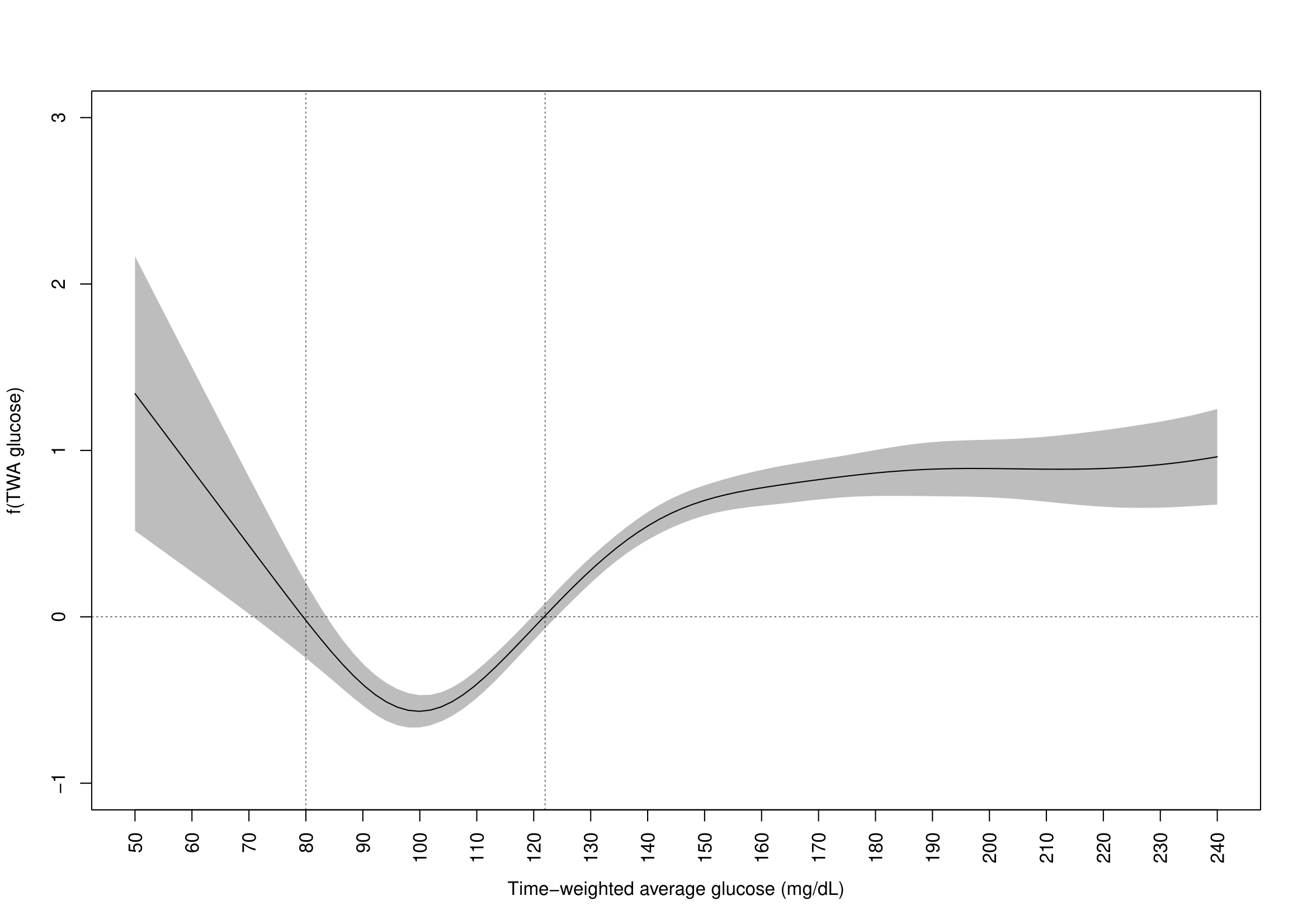


b)


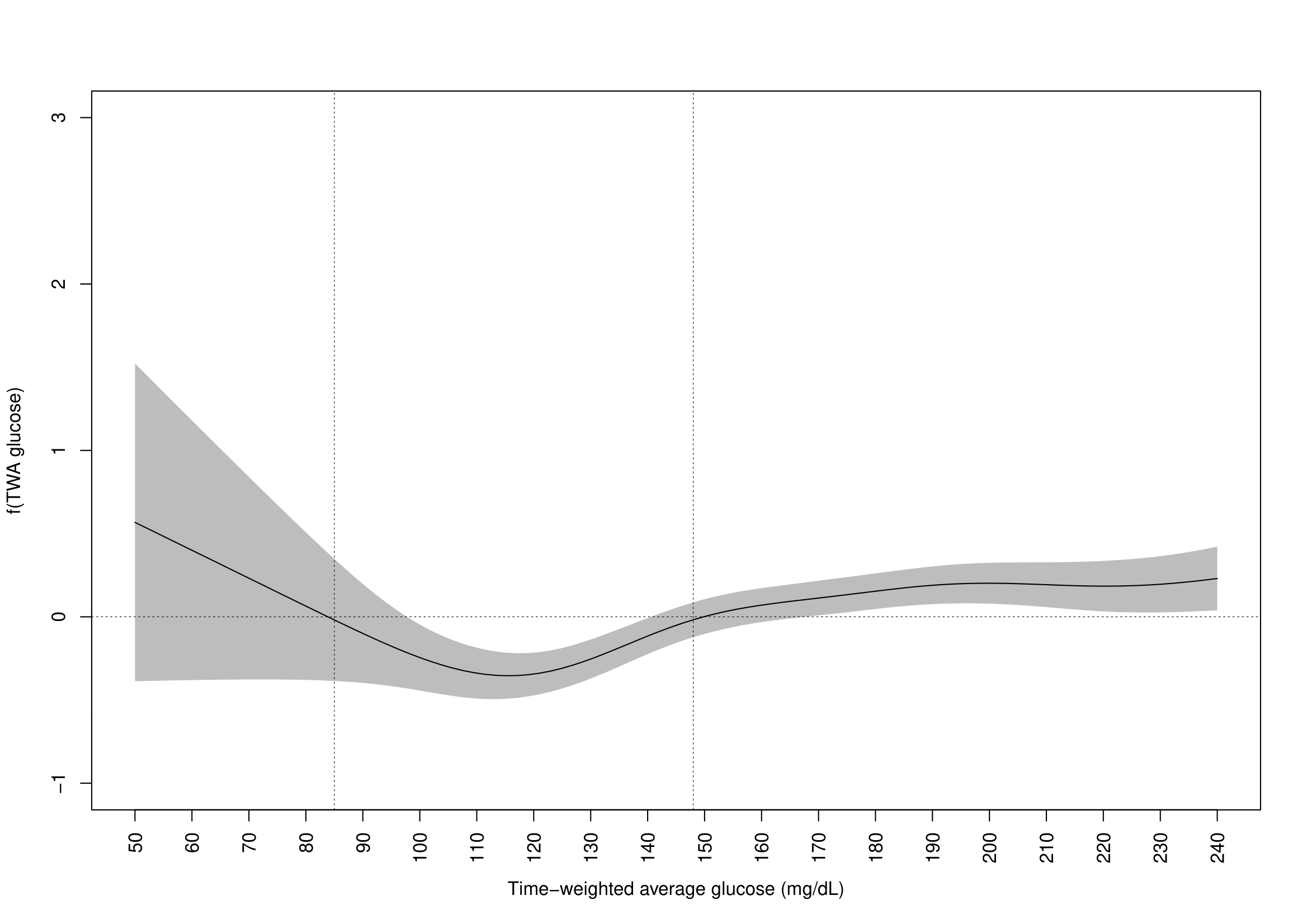


TWA, time weighted average

Association was adjusted for age, APACHE IV scores, body mass index, admission diagnosis, mechanical ventilation, and use of vasopressor or inotropic agents. Cutoffs were obtained where f(TWA glucose)=0 (which represented the average risk). A TWA glucose level ranging approximately from 80-120mg/dL and 90-150mg/dL represented a below-average risk of hospital mortality for patients with no diabetes and patients with diabetes respectively.

Supplementary figure 3. Probability of hospital mortality and time weighted average glucose in medical and surgical patients


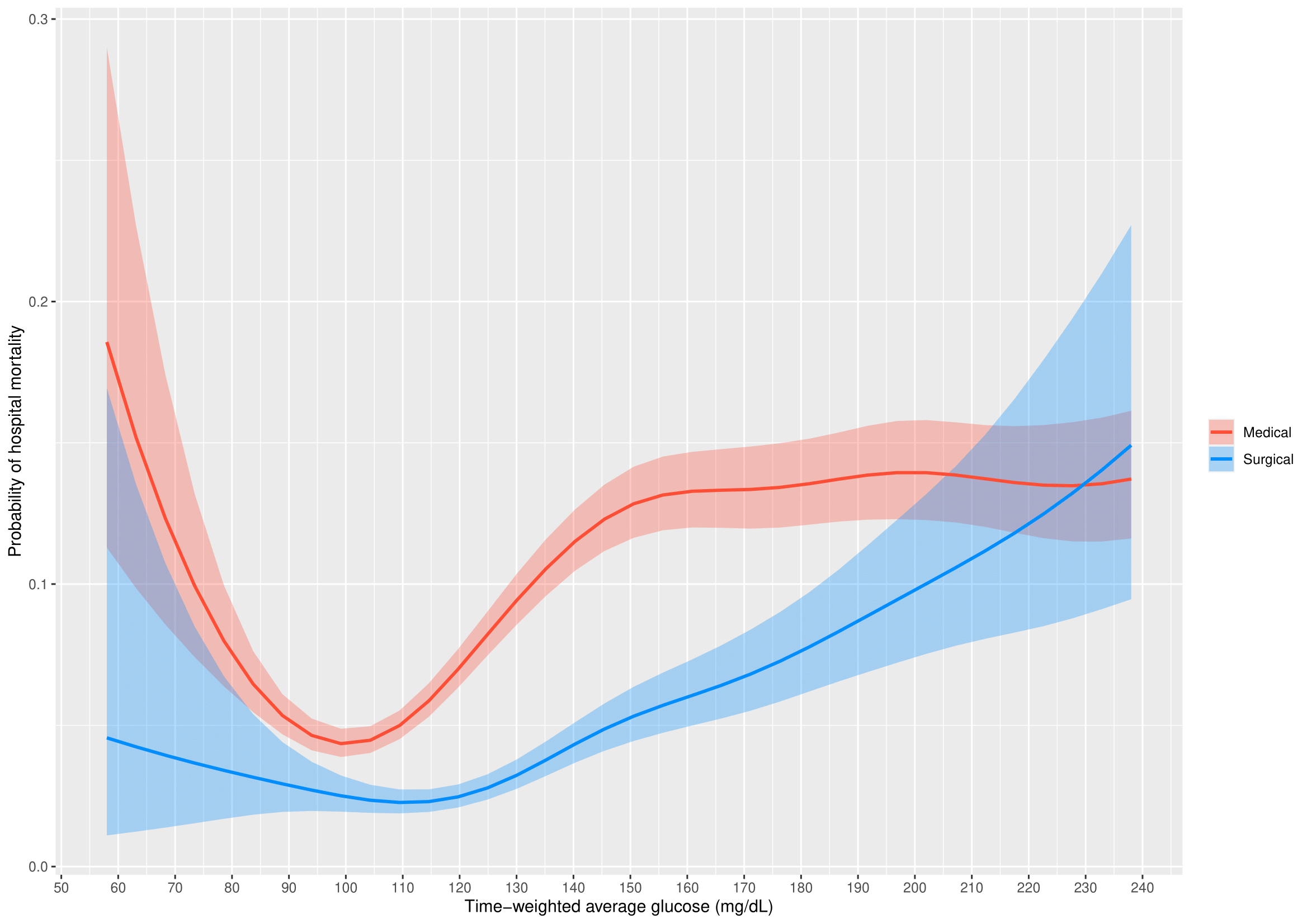


Analysis was adjusted for age, APACHE IV scores, body mass index, admission diagnosis, diabetes, mechanical ventilation, and use of vasopressor or inotropic agents.

Supplementary figure 4. Probability of hospital mortality and minimum glucose in medical and surgical patients


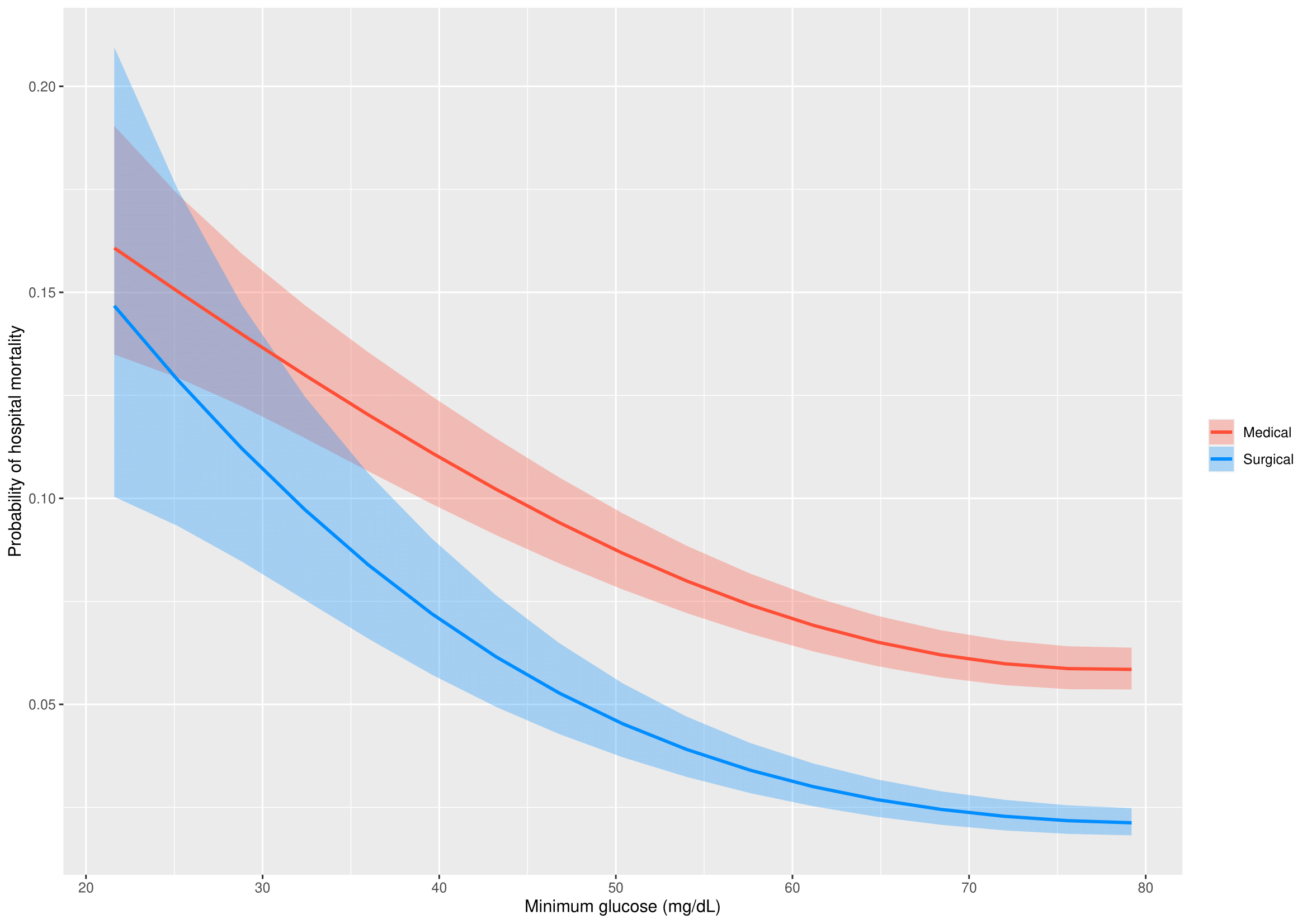


Analysis was adjusted for age, APACHE IV scores, body mass index, admission diagnosis, diabetes, mechanical ventilation, and use of vasopressor or inotropic agents.

Supplementary figure 5. Probability of hospital mortality and coefficient of variation in medical and surgical patients


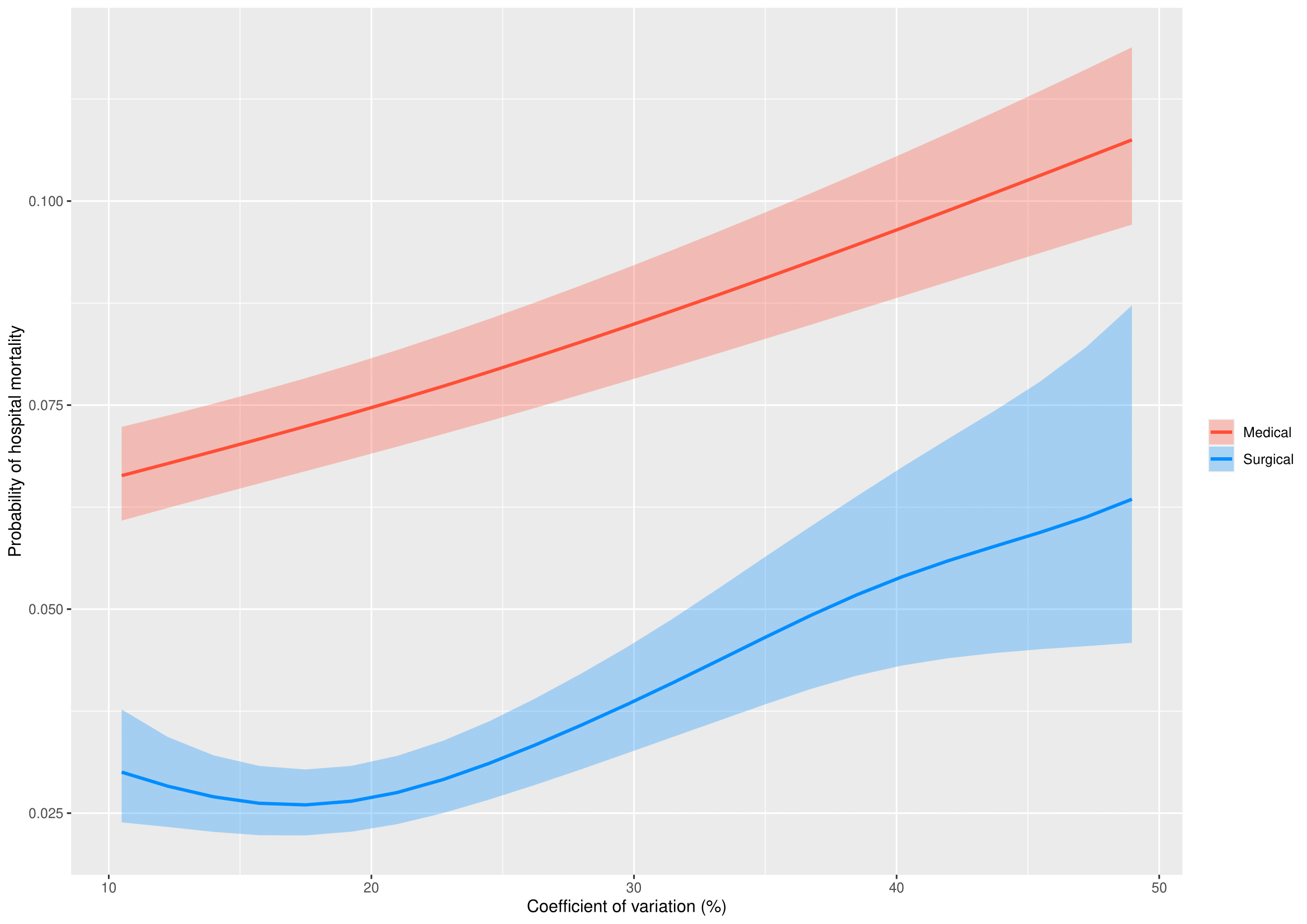


Analysis was adjusted for age, APACHE IV scores, body mass index, admission diagnosis, diabetes, mechanical ventilation, and use of vasopressor or inotropic agents.

Supplementary figure 6. Probability of hospital mortality and time weighted average glucose in trauma and nontrauma patients


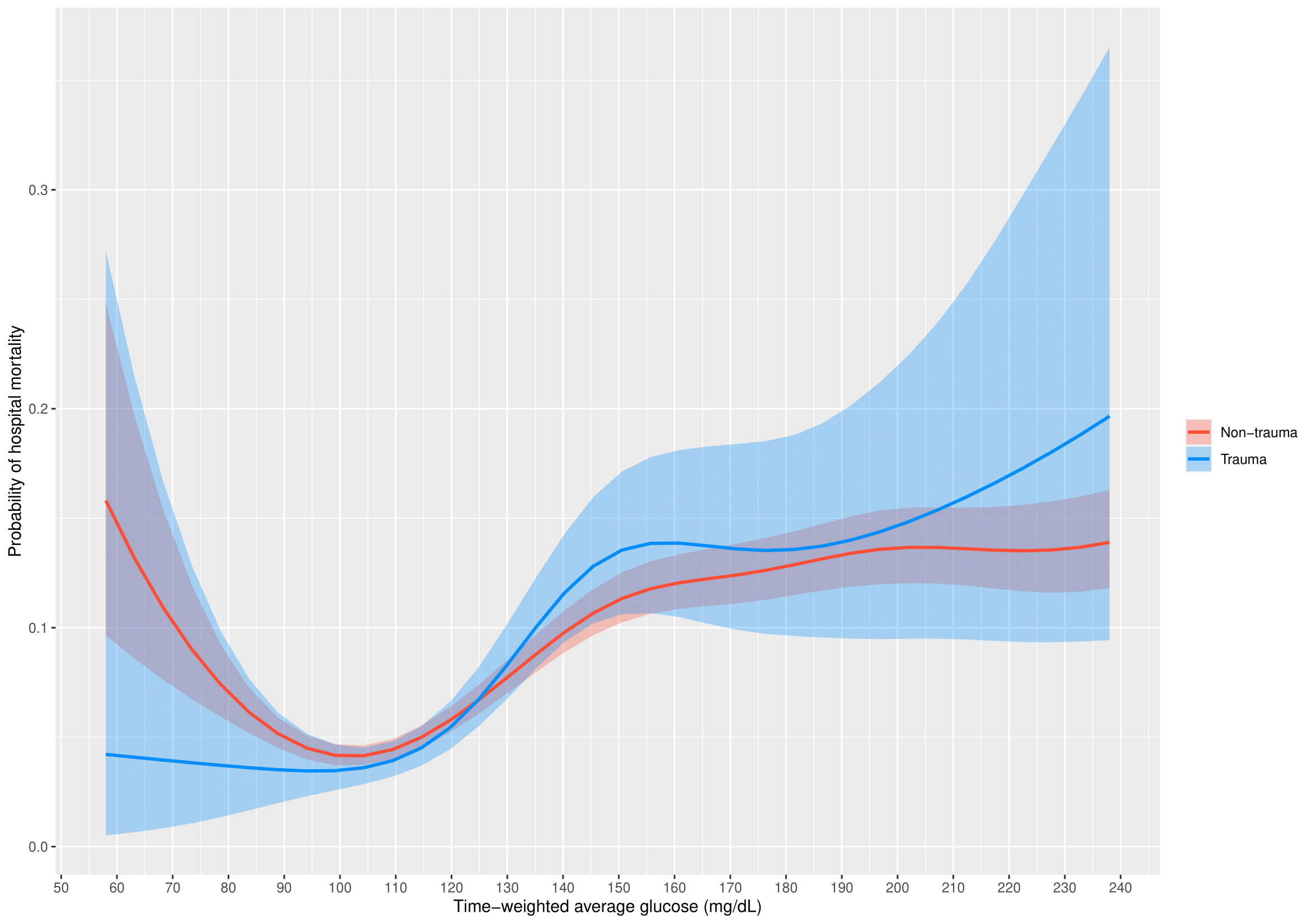


Analysis was adjusted for age, APACHE IV scores, body mass index, diabetes, mechanical ventilation, and use of vasopressor or inotropic agents.

Supplementary figure 7. Probability of hospital mortality and minimum glucose in trauma and nontrauma patients


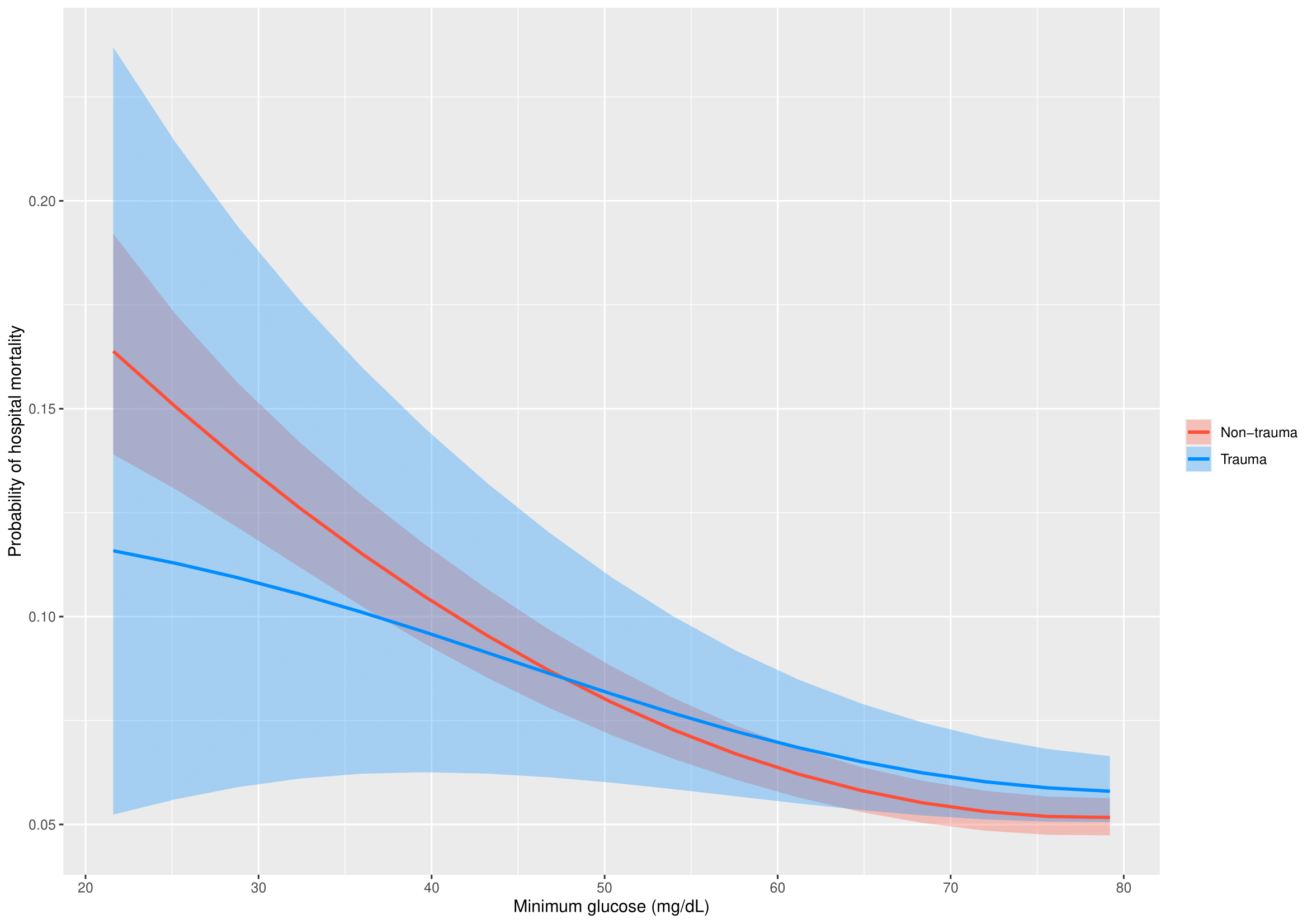


Analysis was adjusted for age, APACHE IV scores, body mass index, diabetes, mechanical ventilation, and use of vasopressor or inotropic agents.

Supplementary figure 8. Probability of hospital mortality and coefficient of variation in trauma and nontrauma patients


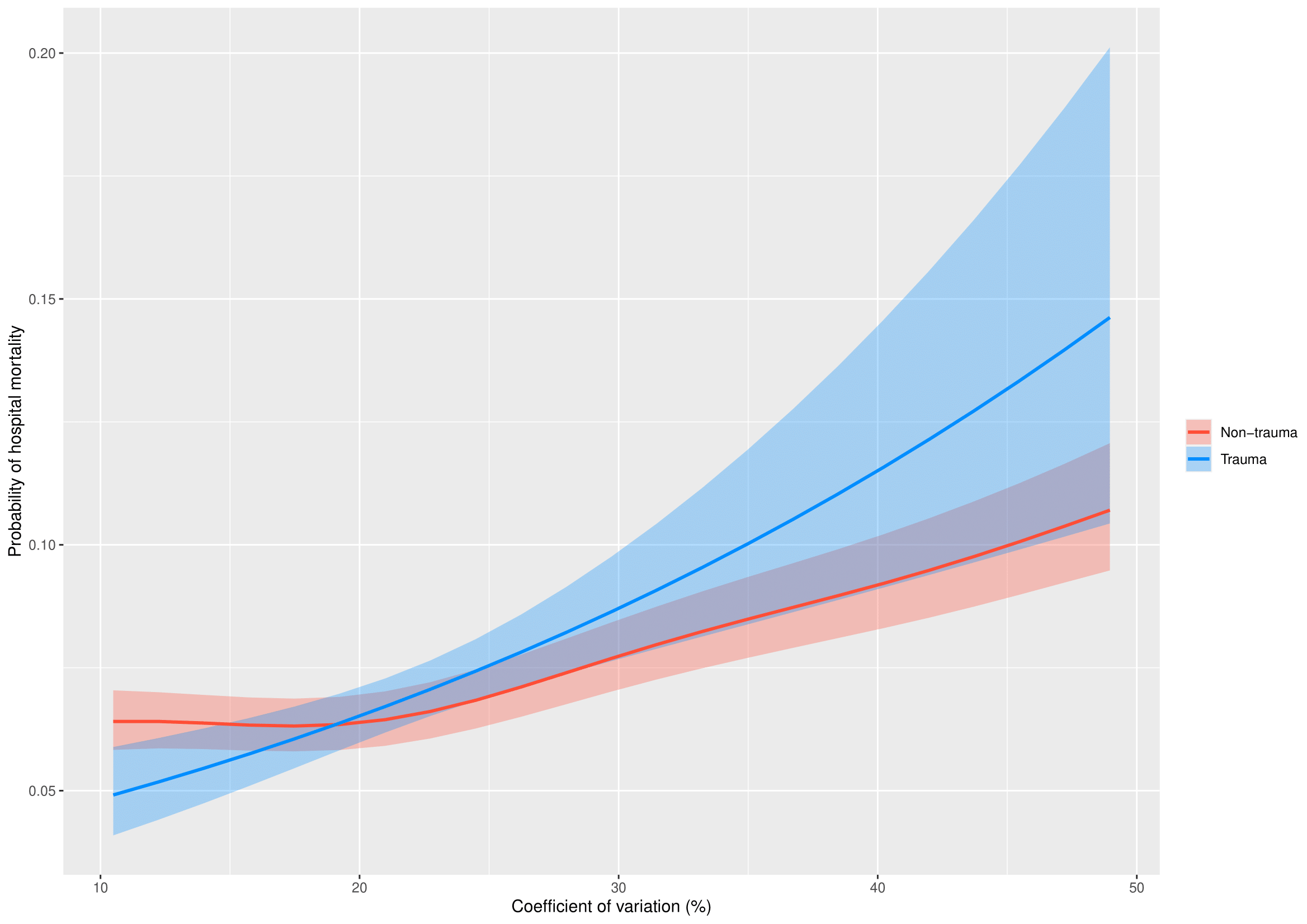


Analysis was adjusted for age, APACHE IV scores, body mass index, diabetes, mechanical ventilation, and use of vasopressor or inotropic agents.

Supplementary figure 9. Probability of hospital mortality and time weighted average glucose in diabetes patients on insulin, oral hypoglycemic agents, or diet and patients with no diabetes


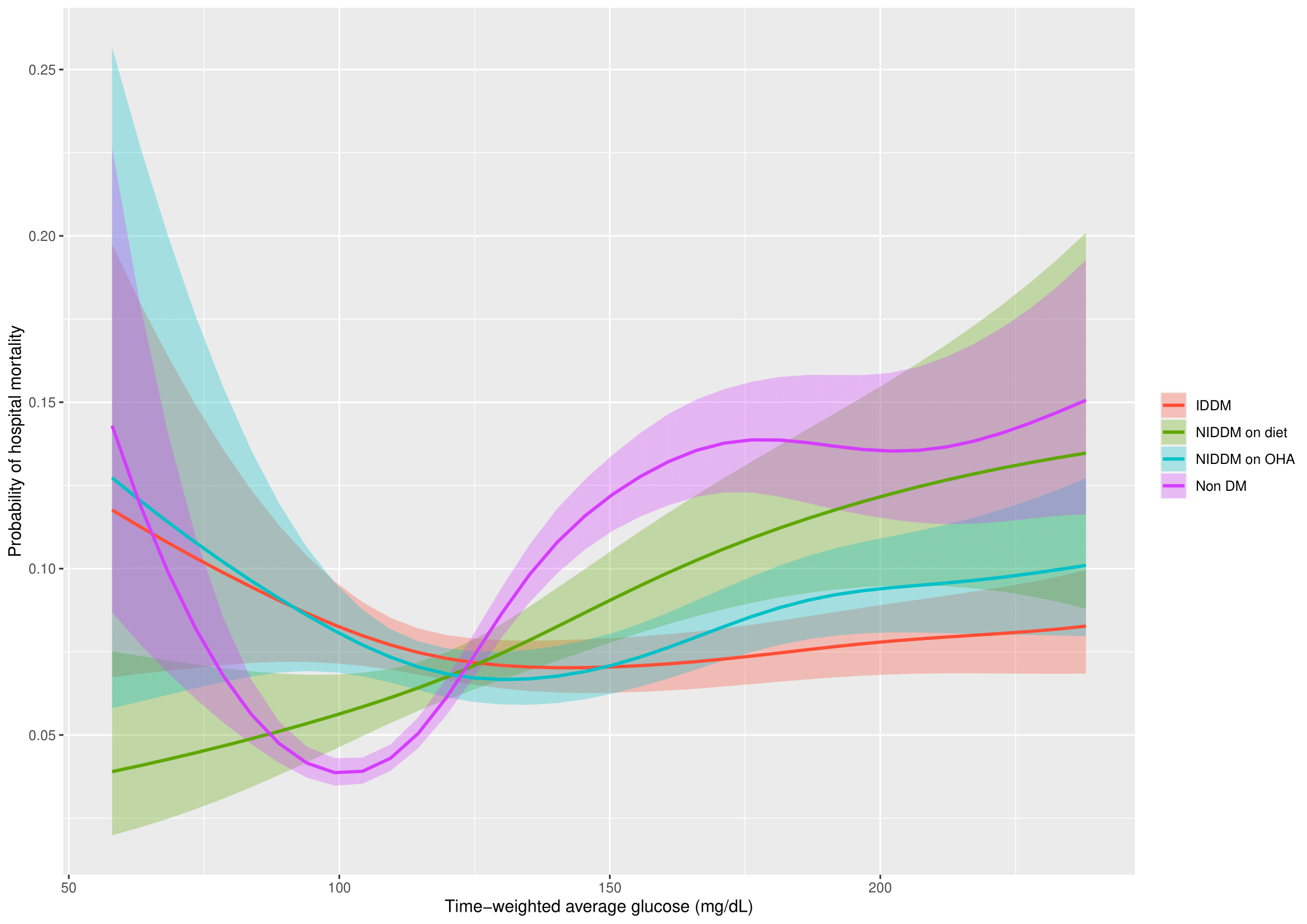


DM, diabetes mellitus; IDDM, insulin-dependent diabetes mellitus; NIDDM, non-insulin dependent diabetes mellitus; OHA, oral hypoglycemic agents;

Analysis was adjusted for age, APACHE IV scores, body mass index, admission diagnosis, mechanical ventilation, and use of vasopressor or inotropic agents.

Supplementary figure 10. Probability of hospital mortality and minimum glucose in diabetes patients on insulin, oral hypoglycemic agents, or diet and patients with no diabetes


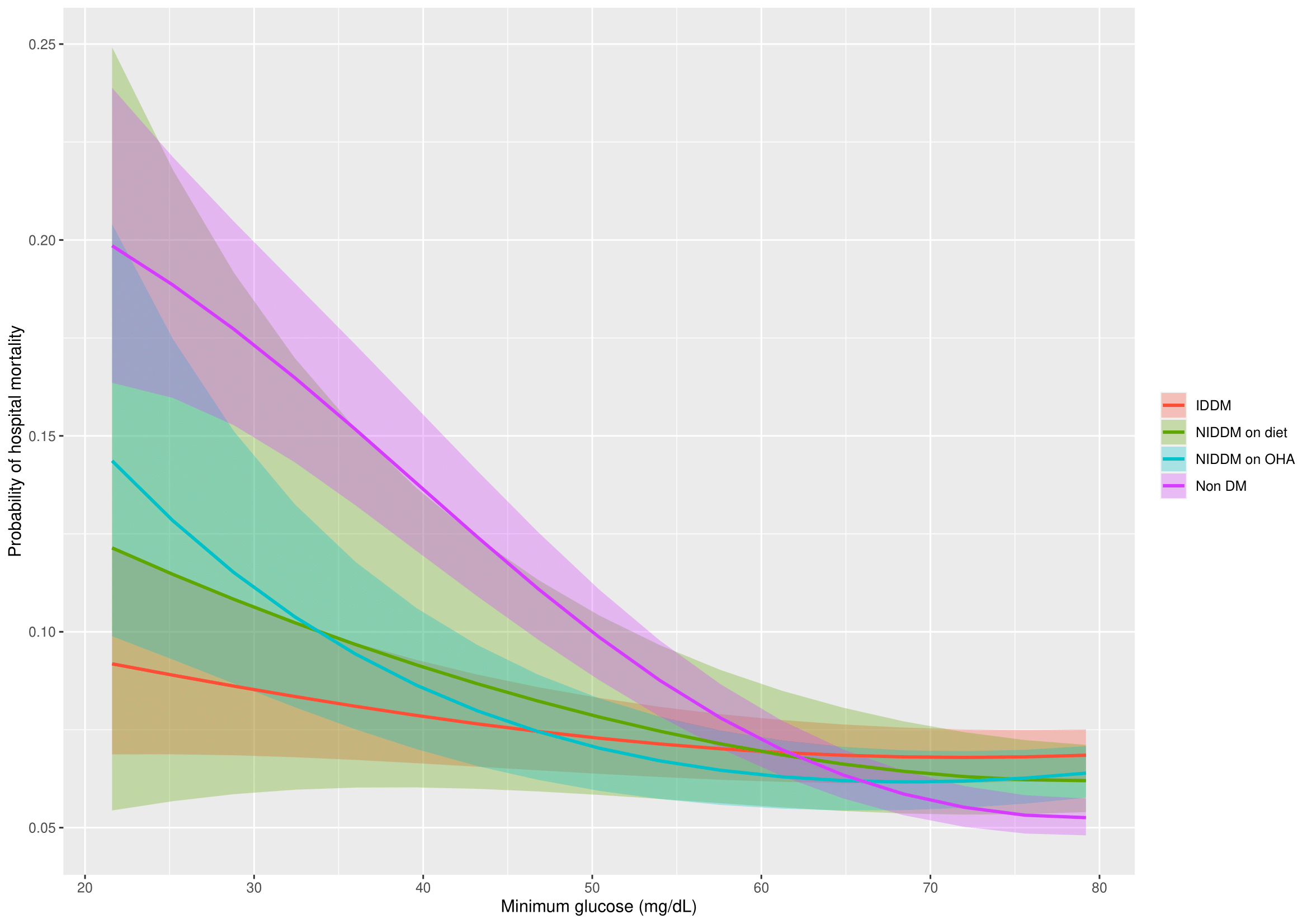


DM, diabetes mellitus; IDDM, insulin-dependent diabetes mellitus; NIDDM, non-insulin dependent diabetes mellitus; OHA, oral hypoglycemic agents;

Analysis was adjusted for age, APACHE IV scores, body mass index, admission diagnosis, mechanical ventilation, and use of vasopressor or inotropic agents.

Supplementary figure 11. Probability of hospital mortality and coefficient of variation in diabetes patients on insulin, oral hypoglycemic agents, or diet and patients with no diabetes


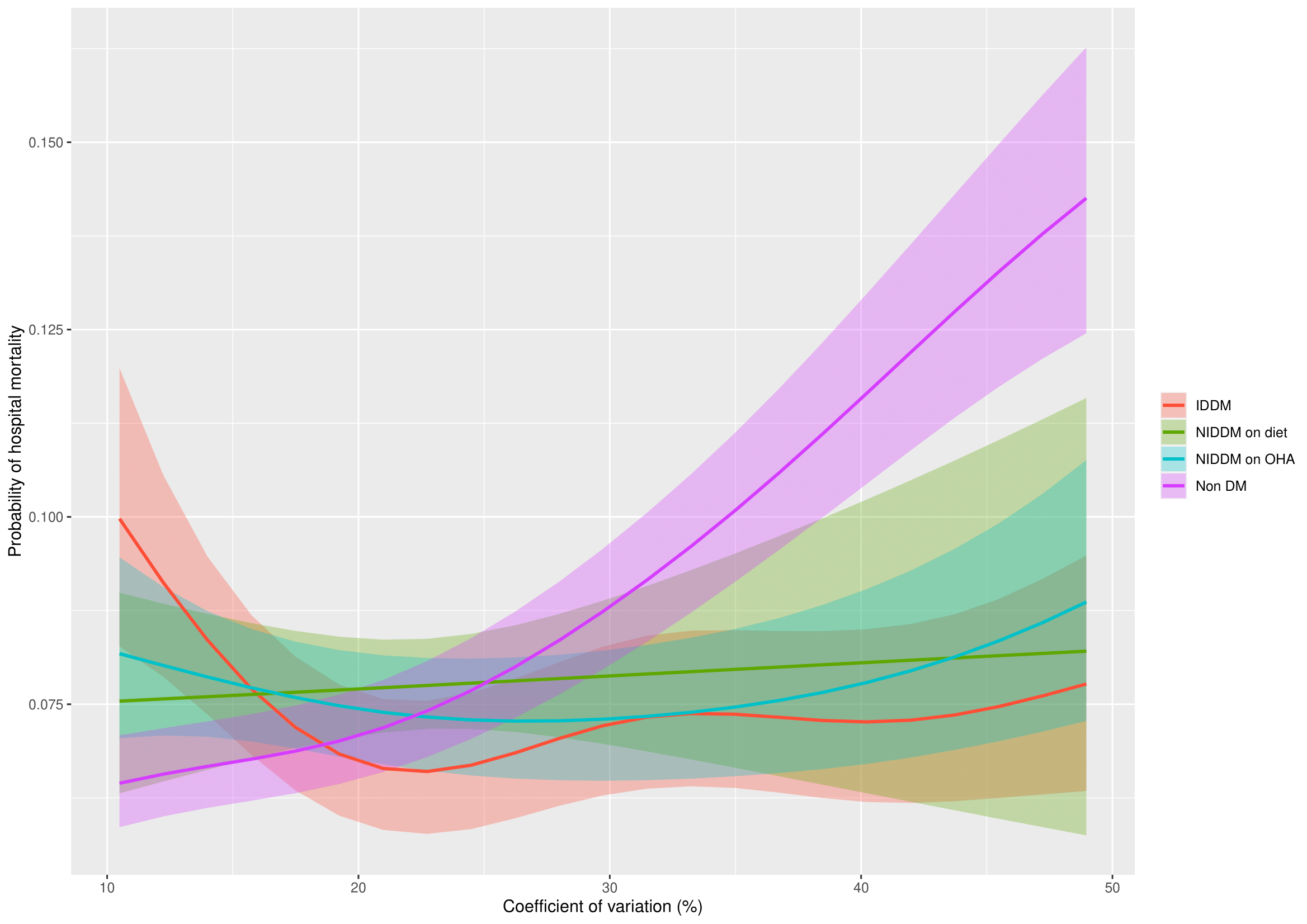


IDDM, insulin-dependent diabetes mellitus; NIDDM, non-insulin dependent diabetes mellitus; OHA, oral hypoglycemic agents; DM, diabetes mellitus

Analysis was adjusted for age, APACHE IV scores, body mass index, admission diagnosis, mechanical ventilation, and use of vasopressor or inotropic agents.

Supplementary figure 12. Probability of hospital mortality and time weighted average glucose in all diabetes and nondiabetes patients (including length of stay < 2 days)


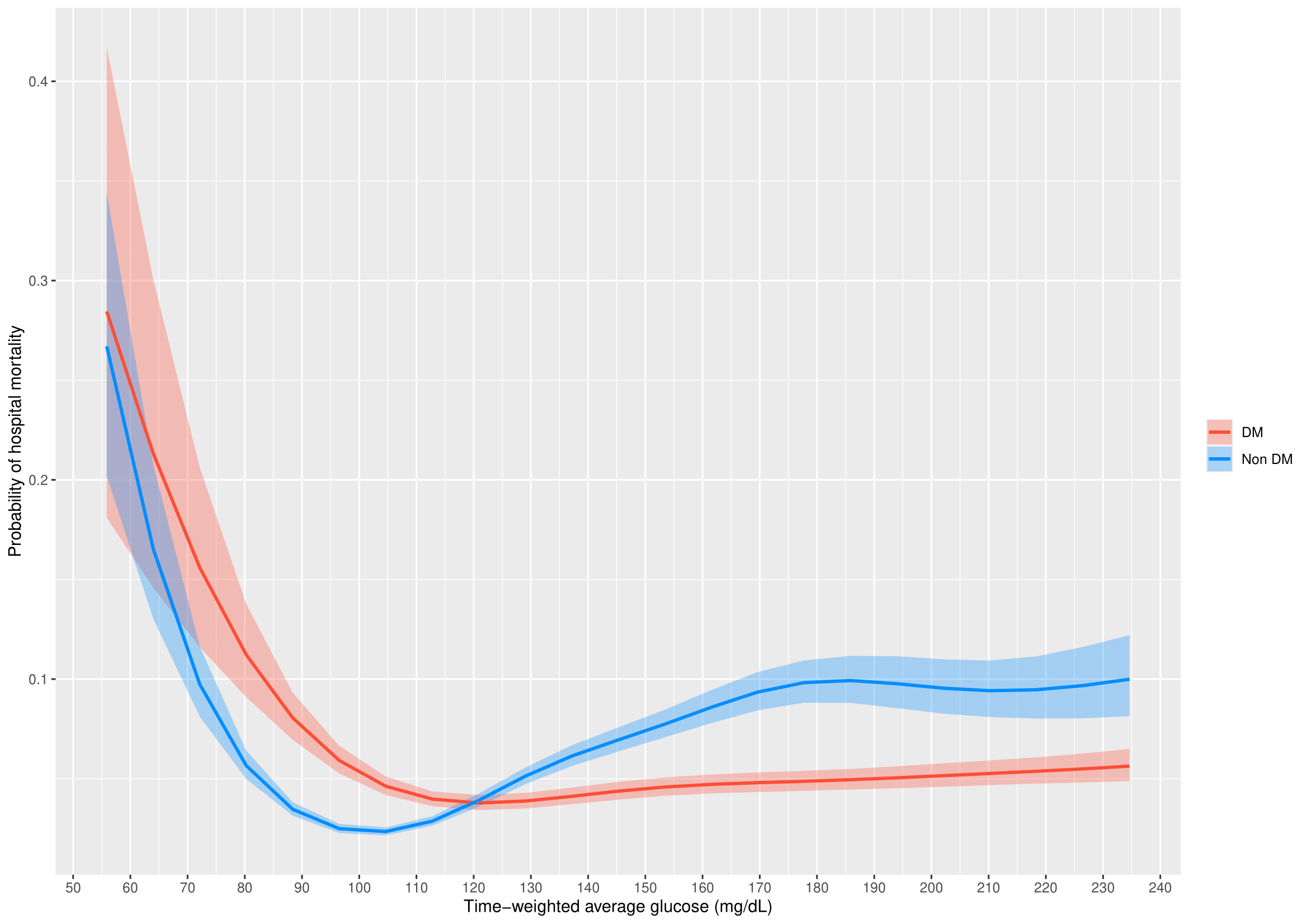


Analysis was adjusted for age, APACHE IV scores, body mass index, admission diagnosis, diabetes, mechanical ventilation, and use of vasopressor or inotropic agents.

Supplementary figure 13. Probability of hospital mortality and minimum glucose in all diabetes and nondiabetes patients (including length of stay < 2 days)


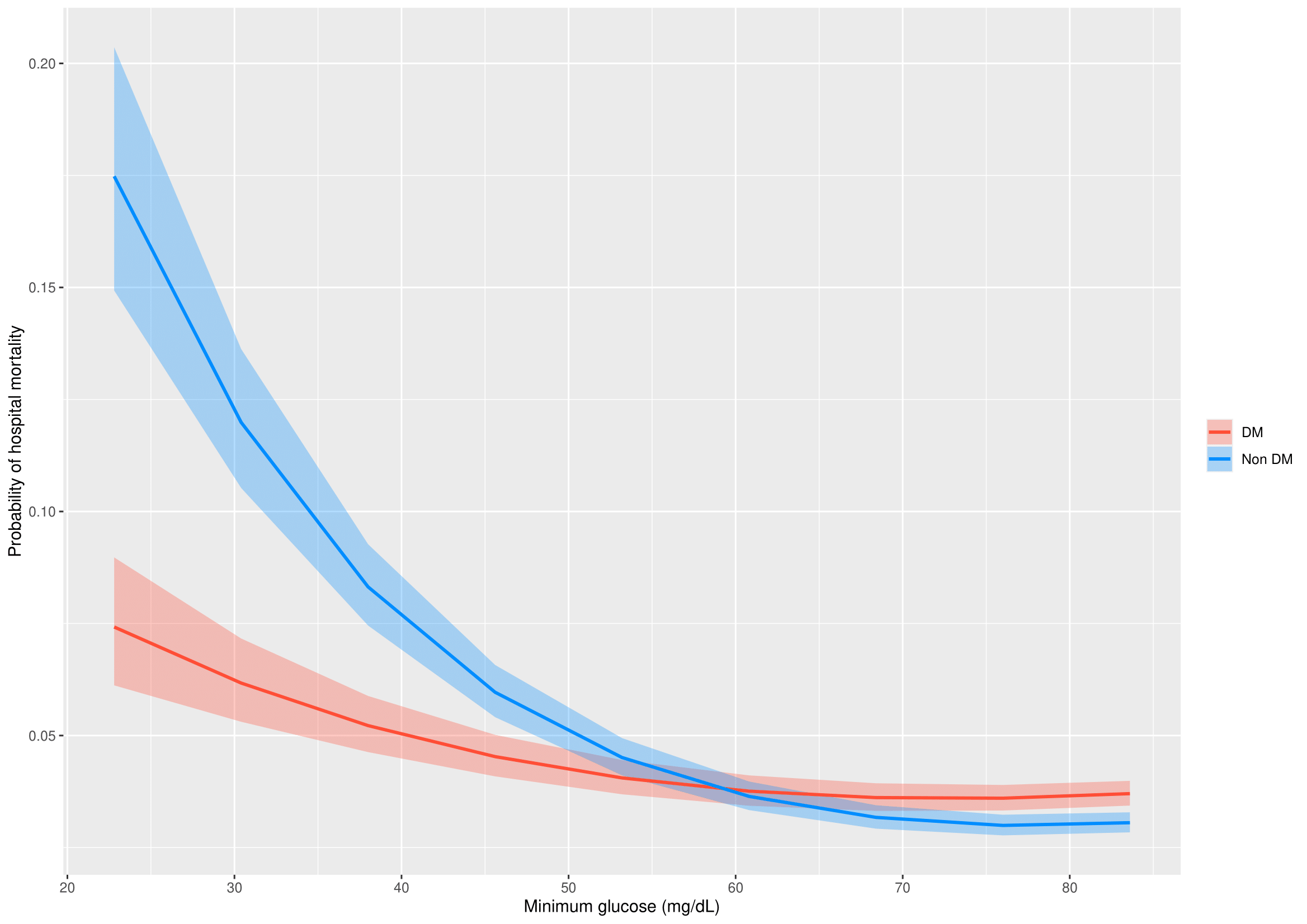


Analysis was adjusted for age, APACHE IV scores, body mass index, admission diagnosis, diabetes, mechanical ventilation, and use of vasopressor or inotropic agents.

Supplementary figure 14. Probability of hospital mortality and coefficient of variation in all diabetes and nondiabetes patients (including length of stay < 2 days)


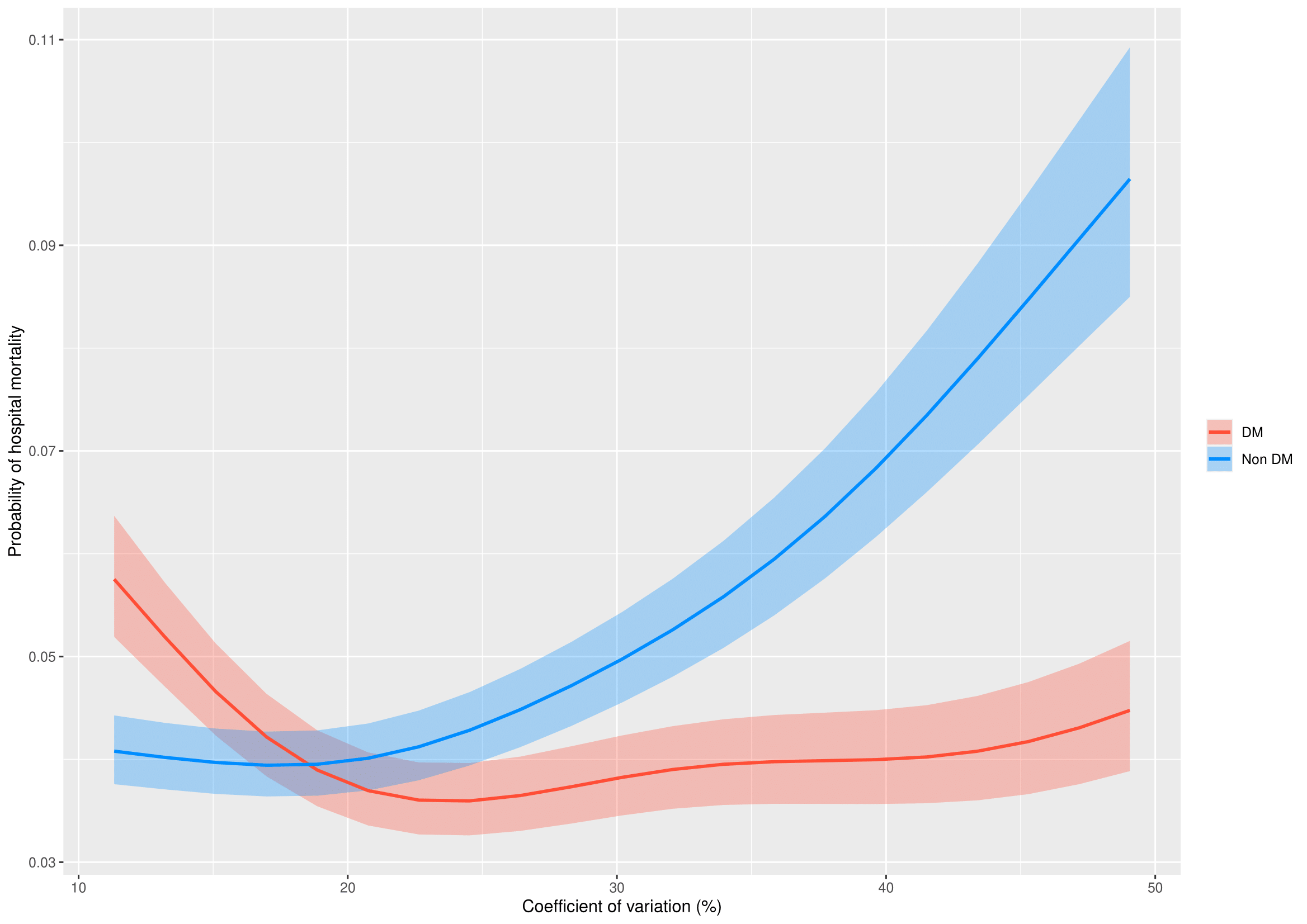


Analysis was adjusted for age, APACHE IV scores, body mass index, admission diagnosis, diabetes, mechanical ventilation, and use of vasopressor or inotropic agents.

Supplementary figure 15. Smoothed scaled Schoenfeld residual plot of glucose on ICU mortality


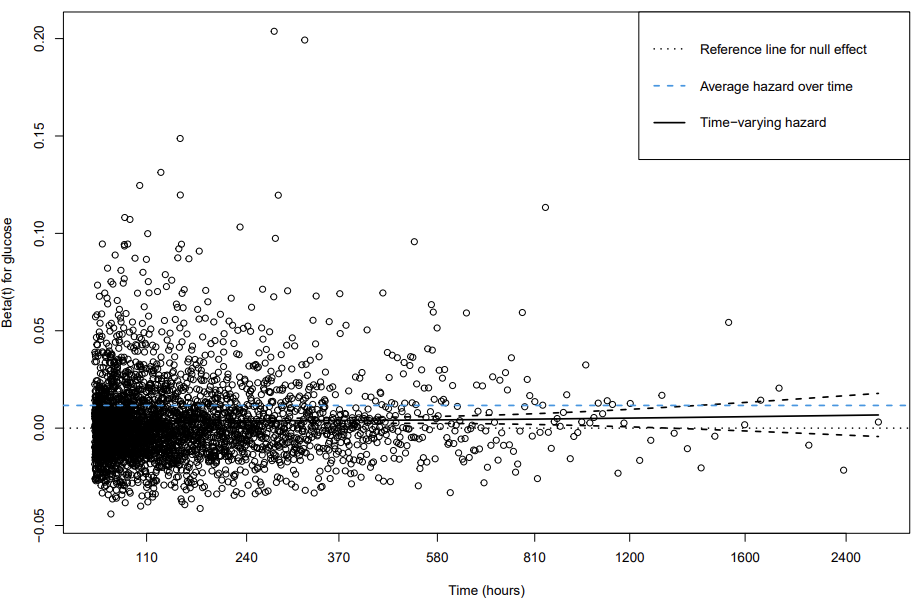

Supplement: Supplementary file 1 — Additional file 1: Supplementary Table 1. Measures of glycemic control. Supplementary Table 2. Number of nondiabetes and diabetes patients on insulin, oral hypoglycemic agents, and diet. Supplementary Table 3. Top ten admission diagnoses in patients admitted for medical or surgical diagnoses. Supplementary Table 4. Odds ratios for the association of hospital mortality on glycemic measures derived from generalised additive model. Supplementary Table 5. Hazard ratios from the Cox proportional hazard model with glucose as time-varying covariate on ICU mortality. Supplementary Table 6. Schoenfeld’s global and individual test for the violation of proportional assumptions of Cox proportional hazard model. Supplementary Figure 1. Patient flow chart. Supplementary Figure 2. Graphical representation of the generalised additive model showing the time weighted average glucose associated with below-average risk of mortality for a) patients with no diabetes and b) patients with diabetes. Supplementary Figure 3. Probability of hospital mortality and time weighted average glucose in medical and surgical patients. Supplementary Figure 4. Probability of hospital mortality and minimum glucose in medical and surgical patients. Supplementary Figure 5. Probability of hospital mortality and coefficient of variation in medical and surgical patients. Supplementary Figure 6. Probability of hospital mortality and time weighted average glucose in trauma and nontrauma patients. Supplementary Figure 7. Probability of hospital mortality and minimum glucose in trauma and nontrauma patients. Supplementary Figure 8. Probability of hospital mortality and coefficient of variation in trauma and nontrauma patients. Supplementary Figure 9. Probability of hospital mortality and time weighted average glucose in diabetes patients on insulin, oral hypoglycemic agents, or diet and patients with no diabetes. Supplementary Figure 10. Probability of hospital mortality and minimum glucose in diabetes patients on [file 12871_2022_1769_MOESM1_ESM.docx]
